# Microalgae, Seaweeds and Aquatic Bacteria, Archaea, and Yeasts: Sources of Carotenoids with Potential Antioxidant and Anti-Inflammatory Health-Promoting Actions in the Sustainability Era

**DOI:** 10.3390/md21060340

**Published:** 2023-06-01

**Authors:** Paula Mapelli-Brahm, Patricia Gómez-Villegas, Mariana Lourdes Gonda, Antonio León-Vaz, Rosa León, Jennifer Mildenberger, Céline Rebours, Verónica Saravia, Silvana Vero, Eugenia Vila, Antonio J. Meléndez-Martínez

**Affiliations:** 1 Food Colour and Quality Laboratory, Facultad de Farmacia, Universidad de Sevilla, 41012 Sevilla, Spain; 2 Laboratory of Biochemistry, Faculty of Experimental Sciences, Marine International Campus of Excellence and REMSMA, University of Huelva, 21071 Huelva, Spain; patricia.gomez@dqcm.uhu.es (P.G.-V.); antonio.leon@dqcm.uhu.es (A.L.-V.); rleon@uhu.es (R.L.); 3 Área Microbiología, Departamento de Biociencias, Facultad de Química, Universidad de la República, Gral Flores 2124, Montevideo 11800, Uruguay; mgonda@fq.edu.uy (M.L.G.); svero@fq.edu.uy (S.V.); 4 Møreforsking AS, Borgundveien 340, 6009 Ålesund, Norway; jennifer.mildenberger@moreforsking.no (J.M.); celine.rebours@moreforsking.no (C.R.); 5 Departamento de Bioingeniería, Facultad de Ingeniería, Instituto de Ingeniería Química, Universidad de la República, Montevideo 11300, Uruguay; vsaravia@fing.edu.uy (V.S.); mvila@fing.edu.uy (E.V.)

**Keywords:** bioactives, natural pigments, antioxidant activity, marine organisms, human health, blue economy, agro-food, functional foods, marine resources, cosmetics

## Abstract

Carotenoids are a large group of health-promoting compounds used in many industrial sectors, such as foods, feeds, pharmaceuticals, cosmetics, nutraceuticals, and colorants. Considering the global population growth and environmental challenges, it is essential to find new sustainable sources of carotenoids beyond those obtained from agriculture. This review focuses on the potential use of marine archaea, bacteria, algae, and yeast as biological factories of carotenoids. A wide variety of carotenoids, including novel ones, were identified in these organisms. The role of carotenoids in marine organisms and their potential health-promoting actions have also been discussed. Marine organisms have a great capacity to synthesize a wide variety of carotenoids, which can be obtained in a renewable manner without depleting natural resources. Thus, it is concluded that they represent a key sustainable source of carotenoids that could help Europe achieve its Green Deal and Recovery Plan. Additionally, the lack of standards, clinical studies, and toxicity analysis reduces the use of marine organisms as sources of traditional and novel carotenoids. Therefore, further research on the processing of marine organisms, the biosynthetic pathways, extraction procedures, and examination of their content is needed to increase carotenoid productivity, document their safety, and decrease costs for their industrial implementation.

## 1. Introduction: Sustainability, the Blue Economy, and the Versatility of Carotenoids in Agro-Food and Health

It is estimated that 40% of the global land surface is used by agriculture and that 30% of global greenhouse gas emissions and 70% of freshwater use are related to food production. Besides, 820 million people have insufficient food, 2000 million adults are overweight or obese, and the global prevalence of diabetes has doubled since 1990 [1]. Thus, research on food science, technology, and related fields such as agriculture, aquaculture, and nutrition must be directed toward the sustainable production of health-promoting foods to combat diet-related diseases and preserve key resources for future generations. The synergistic work carried out in large related networks (CYTED-IBERCAROT, CYTED-MICROAGRO, COST-EUROCAROTEN, COST-SEAWHEAT, COST-OCEAN4BIOTECH, CaRed, etc.) is important to orchestrate efforts and produce breakthroughs.

The rapid growth of the global population requires new solutions to ensure food security, which cannot rely exclusively on land-based agriculture; hence, the importance of tapping further into the vast aquatic biodiversity. The blue economy of the EU is fundamental for the European Green Deal and the Recovery Plan for Europe, which will define the European economy for many years, as the ocean is the main climate regulator, offers clean energy, and provides us with oxygen and food, among other resources. The Blue Economy includes all those activities that are marine-based or marine-related and encompasses sectors including living and non-living resources or renewable energy, among others. Important marine species featured in the blue bioeconomy are algae (microalgae and seaweeds), bacteria, fungi, and invertebrates. Each year, hundreds of untapped new compounds derived from these organisms are discovered, including carotenoids and their derivatives. The applications of their biomasses in agro-food health range from foods, supplements, cosmetics, and feeds to biostimulants and fertilizers. They can also be used for biomaterials, bioremediation, or biofuels [2]. Regarding the carotenoids, the Action COST EUROCAROTEN (www.eurocaroten.eu (accessed on 30 May 2023)) highlighted that apart from marine sources, understudied carotenoids (the great majority, as only 10–20 carotenoids are being studied in depth) offer many possibilities for innovation in agro-food, health, cosmetics, or biomaterials [3].

Algae and microbes (as well as insects) are highlighted as matrices that can be further harnessed to produce innovations in the context of healthy diets from sustainable food systems in the EAT-Lancet Commission report [1]. Microalgae and macroalgae received much interest as biofuel feedstocks in response to the uprising energy crisis, climate change, and the depletion of natural sources. Concomitantly, high-value co-products, including carotenoids, have been produced through the extraction of a fraction of algae to improve the economics of a microalgae biorefinery. This is the process of obtaining biofuels, energy, and diverse high-value products through biomass transformation and process equipment [4,5]. The advantages of using algae biomass as feedstock to produce biofuels and co-products compared to higher plants are several: higher growth and productivity due to their all-year production capability; growth under stress conditions and lower nutritional and water requirements; no requirements of herbicides or pesticides; contribution to CO_2_ sequestration and wastewater bioremediation; easier modulation of the biosynthesis of valuable co-products by modifying growth conditions [5,6,7]. It is no wonder that the importance of algae (either as whole cells or for the extraction of high-value components) for innovation in agro-food was highlighted in recent reports from the European Commission and the Food and Agriculture Organization (FAO) [8,9]. More than 20 genera of cyanobacteria and microalgae are currently used for food or feed applications. This number is expected to increase considerably over the next few years as research on such microalgal applications grows incessantly [8]. Regarding macroalgae, some species are exploited and used for human consumption in Europe, particularly in France, Spain, and Ireland, where several companies harvest edible seaweed. These new types of industries have recently been developed following the increasing demand from European consumers [10]. Recent reviews highlight the incorporation of algae (either whole cells or extracts) into a wide variety of foods (emulsions, vegetarian gels, dairy products, cookies, bread, and pasta) with potential health-related and even technofunctional benefits [6,11]. 

Being microbes, bacteria share biotechnological and sustainability advantages with microalgae. Indeed, they are similarly important for alleviating the greenhouse effect, as they can also assimilate CO_2_ [12]. Bacteria have long been used for food production in diverse types of fermentation to obtain food components (including carotenoids, as discussed later) [13,14] and in the last decades, they have attracted great interest in the context of the development of probiotics and the role of the microbiome on human health [15,16,17]. Currently, there is a growing trend to look for innovative applications in aquaculture [18]. 

**Figure 1:**
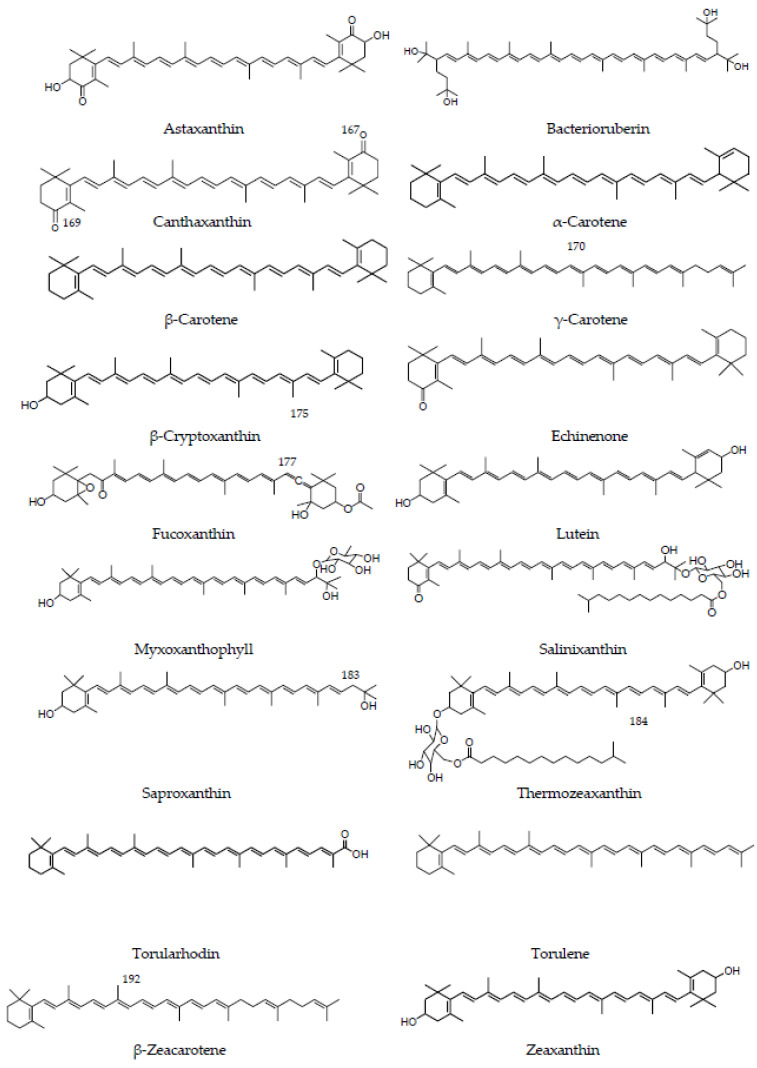
Chemical structures of the main carotenoids present in marine organisms.

Marine archaea offer many opportunities for innovation as they produce unique metabolites as a result of their adaptation to extreme environments. Examples are the C45 carotenoid dihydroisopentenyldehydrorhodopin, the C50 carotenoid bacterioruberin, or the glycoside esters thermozeaxanthin and salinixanthin (Figure 1), whose structures (and therefore their physico-chemical properties) differ greatly relative to typical dietary carotenoids [19].

Yeasts are ubiquitous. Marine yeasts were first discovered in the Atlantic Ocean, and since then they have been isolated from seawater, marine deposits, seaweed, fish, marine mammals, and sea birds. They have also been found in benthic animals and seafloor sediment at depths up to 11,000 m. They are more abundant in nearshore habitats (10–1000 cells/L of water), whereas in low organic surface to deep-sea oceanic regions, they are found at densities <10 cells/L [20]. Some of the many marine yeast genera are *Rhodotorula*, *Rhodosporidium*, *Candida*, *Cryptococcus*, *Torulopsis*, and *Saccharomyces* [21].

The ability of algae, bacteria, and yeasts to use diverse products for growing (especially agriculture and aquaculture wastes or even flue gases in the case of algae) makes them very attractive in the context of the circular economy [22,23,24,25,26,27,28,29,30,31].

Carotenoids are unsaturated compounds that can be classified into two types: carotenes, which are hydrocarbons, and xanthophylls, which present one or more functional groups containing oxygen [32]. Beyond their long-known roles as natural colorants and (some of them) precursors of vitamin A, a large body of evidence indicates that they (or their derivatives, commonly known as apocarotenoids) may be involved in health-promoting biological actions, contributing to reducing the risk of non-communicable diseases as well as attracting renewed interest in cosmetic products [33,34,35]. According to the last update of the carotenoid database on 1 November 2020 (http://carotenoiddb.jp, accessed on 30 May 2023), 680 natural carotenoids from different eukaryotic organisms have been described, including animals, plants, algae, filamentous fungi, and yeasts. Various marine organisms can accumulate carotenoids and represent a route of entry for these compounds into the food chain (Figure 1, Tables 1–5). Thus, they can be considered a natural and renewable source for carotenoid production. However, to date, the industrial production of carotenoids has been based primarily on chemical synthesis [29]. Their extraction from marine organisms cannot compete economically with chemical synthesis, due to the high costs associated with their production and with downstream extraction and purification processes. However, it should be taken into account that obtaining the same isomeric mixtures as those found in natural sources could increase the cost of the production process. For example, in the case of astaxanthin, a mixture of stereoisomers is generally obtained by chemical synthesis, while astaxanthin from natural sources is mainly represented by a single isomer and has higher antioxidant activity and stability [36]. In any case, current trends in natural products have renewed interest in the use of microbial carotenoids as an alternative to those chemically produced [37]. This preference is mainly based on safety concerns about the hazardous waste and intermediate products that could be produced in chemical synthesis. The obtention of carotenoids from marine organism cultures is considered a sustainable process by which carotenoids are produced by milder and more environmentally friendly procedures. The extraction of carotenoids from marine organisms also has advantages over the extraction from plants. They have higher growth rates than vegetables, and their production is for most of these organisms not as affected by seasons or geographic variabilities. Most of them are reasonably easy to cultivate and can be produced in large quantities within a limited space. Moreover, some marine organisms, as a source of carotenoids, can be cultivated on agro-industrial waste or by-products in processes supporting the concept of a circular economy.

This review assesses the potential use of marine organisms as a sustainable source of carotenoids, including well-known and novel carotenoids, as a fundamental part of the European Green Deal and its Recovery Plan. In addition, the different challenges that need to be addressed to obtain new carotenoids on an industrial scale are discussed. The possible antioxidant and anti-inflammatory actions of marine carotenoids are also discussed.

## 2. Main Marine Organisms Containing Carotenoids

### 2.1. Archaea

#### 2.1.1. Ecological Importance

Archaea constitute a domain of prokaryotic organisms with riveting characteristics and high ecological importance. This domain was introduced by Carl Woese in 1990 [38] and originally included only extremophilic microorganisms, concretely methanogens, thermophiles, and halophiles. However, members of the domain Archaea are now known to be widespread in non-extreme habitats, including aquatic and terrestrial environments. In marine ecosystems, planktonic archaea are classified into four distinct groups designated Marine Group (MG) from I to IV, with a growing number of phylum, class, order, and family designations [39]. *Thaumarchaeota* (MGI) have an important role in marine biogeochemistry as they perform the oxidation of ammonia to nitrite, the first step of nitrification. In addition, thaumarchaea are capable of synthesizing cobalamin, the precursor of vitamin B12, which is required by other planktonic members that are not capable of producing it [40]. The MGII archaea are a diverse group of uncultivated heterotrophic and photoheterotrophic microbes with a potential role in the ocean carbon cycle because they are able to break high-molecular-weight compounds. Finally, the MGIII and MGIV archaea are usually found in the deep sea, and so far, little information about them is available [41].

Archaeal communities are also present in freshwater environments, where their main biogeochemical role is related to methanogenesis and ammonium metabolism [42]. Methanogenic archaea, including *Methanobacteriales*, *Methanosarcinales*, and *Methanomicrobiales*, are usually found in anaerobic water and sediments, while ammonia-oxidizing archaea, such as *Thaumarchaeota* and *Nitrosotalea-like*, are often present in oligotrophic surface freshwaters. In addition, some potential genome-based roles, including fermentative metabolism or symbiosis, are being studied in *Woesearchaeota*, the main group found in boreal lakes [43].

The most studied archaea are extremophiles distributed in aquatic environments under severe conditions. Halophiles inhabit salt lakes, soda lakes, and salterns, while hyperthermophiles live in hot springs and shallow or deep hydrothermal systems. Among them, methanogens are represented in halophiles, thermophiles, psychrophiles, acidophiles, and alkaliphiles [44]. Many hyperthermophilic archaea are sulfur-chemolithoautotrophs (e.g., *Sulfolobus*), and complete denitrification is usual in some families of haloarchaea such as *Haloarculaceae* and *Haloferacaceae* [45].

#### 2.1.2. Sustainability Pros and Cons

Archaea are relevant microorganisms for the sustainable production of carotenoids. Currently, bacterioruberin is one of the few products commercialized from archaea [46], and its bioprocess production ensures a low environmental footprint by re-using raw materials in the fermentation cycles [47]. From a biotechnological point of view, the extreme conditions in which haloarchaea proliferate reduce the risk of culture contamination by other microorganisms and allow their cultivation under non-sterile conditions with reduced cultivation costs. Additionally, given that haloarchaea undergo cell lysis at low concentrations of salt, carotenoids can be easily recovered under mild conditions with lower costs than from other microorganisms that require harsh cell disruption methods. Finally, haloarchaea show an advantage over other microorganisms as a source of bioactive products that can be used in personal care products, drugs, or foods, as they are non-pathogenic for animals and humans [19].

#### 2.1.3. Main Species (and Compounds) in the Context of Agro-Food and Health

Archaea are a valuable source of unique carotenoids with promising applications in cosmetics, food, pharmaceuticals, and nutraceuticals. To date, 25 different carotenoids and apocarotenoids have been described in Archaea, 13 of which are only present in this domain. Most archaeal carotenoids are synthesized by haloarchaea, with bacterioruberin being the most abundant [19] (Table 1, Figure 1).

Bacterioruberin is an unusual C50 carotenoid whose function is related to UV protection and membrane stability. The complete biosynthetic pathway of bacterioruberin from lycopene was elucidated in *Haloacula japonica* by Yang et al. [55], and intermediates such as monoanhydrobacterioruberin and bisanhydrobacterioruberin are usually present in different proportions in haloarchaeal extracts [49,50,51]. Bacterioruberin possesses 13 carbon double bonds and 4 hydroxyl groups at the terminal ends that confer it a high antioxidant potential, even greater than the reference antioxidant β-carotene under certain conditions. Several studies have demonstrated the antioxidant activity of bacterioruberin-rich extracts [51,56], and some others have also evaluated the different bioactivities of haloarchaeal pigments. In this context, carotenoids extracted from *Halobacterium halobium*, *Halogeometricum limi*, and *Haloplanus vescus* have shown antiproliferative activities in the human cancer cell line HepG2 (hepatocarcinoma) [48,57]. In addition, carotenoids from *Natrialba* have shown antiviral activity against viral hepatitis (HCV and HBV) and potent antiproliferative and apoptotic activities against colon, breast, liver, and cervical cancer cells, as well as being safer than the standard anticancer drug 5-FU for normal human cells under certain conditions [58]. Furthermore, bacterioruberin-rich extracts from various species of haloarchaea, including the genera *Haloarcula*, *Halobacterium*, *Halorubrum*, and *Haloferax*, have displayed diverse bioactive properties in vitro, such as antibacterial, neuroprotective, anti-inflammatory, antiglycemic, and antilipidemic activities [52,53,54,59]. Finally, bacterioruberin-enriched extracts from a genetically modified *Haloferax volcanii* strain were shown to improve the quality of cryopreserved ram sperm cells, suggesting their potential application to increase insemination yields [60].

#### 2.1.4. Production Approaches and Production Data in Different Continents

The extreme growth conditions required for extremophilic archaea that synthesize carotenoids have made the development of laboratory strategies for their culture and the production of biocompounds difficult. Studies on the genus *Haloferax* revealed that the bacterioruberin synthesis can be enhanced by adding carbon sources (glucose or starch) to the medium [54] and also by using a culture medium with lower salt (less than 15% *w*/*v*) [61]. Thus, bacterioruberin production from *Haloferax* has been proposed in a two-step process, using a first step with a high salt concentration (20%, *w*/*v*) for biomass production and a second step with lower salt concentrations for the accumulation of carotenoids [62]. However, other species of the genera *Halorubrum* and *Natriabla* required a salt concentration of 20–25% *w*/*v* for the maximum production of carotenoids [50,52,58], and therefore the process could be performed in a single step. Moreover, many other physicochemical parameters have to be taken into account to optimize the carotenoids produced by haloarchaea. In this regard, diverse species of haloarchaea cultured with a nearly neutral pH, a temperature of 30 to 40 °C, high light exposure, and high oxygen availability have experimented with an increase in growth rate and carotenoid productivity. It has also been demonstrated that the amount of MgSO_4_, KCl, and trace elements may influence the production of bacterioruberin [63].

#### 2.1.5. Current and Prospective Applications

Archaeal carotenoids offer a wide range of potential applications in foods, health promotion, and cosmetics, for instance, as nutraceuticals, colorants, or food preservatives. Given the antioxidant and photoprotective properties of bacterioruberin, the possibility of using it as a natural additive for foods and personal care products is quite realistic. The haloarchaeal carotenoid bacterioruberin is commercially produced as “Halorubin” by the company Halotek (Leipzig, Germany) [46].

Despite the bioactive properties described for bacterioruberin, its chemical lability, poor water solubility, and low bioavailability limit many of its potential health-promoting uses. Therefore, the use of nanovesicles (archaeosomes or nanoarchaeosomes) and nanoparticles has been proposed to improve the stability and biological activities of bacterioruberin [64]. In the same way, the encapsulation of archaeal carotenoids into two oil-in-water dispersions has been reported as a useful system for the delivery and protection of carotenoids for food applications [65].

#### 2.1.6. Research Needs

Carotenoids from Archaea have shown considerable potential for health-promoting effects. Nonetheless, the small number of studies on natural products from Archaea compared to Bacteria and Eukarya suggests that this field of research is still relatively underexplored. Considering the extreme environments in which extremophilic archaea reside, it can be assumed that they contain many new molecules of biotechnological interest whose identification is still pending.

The biotechnological applications of bacterioruberin contribute to the increasing demand for this carotenoid. Nowadays, commercial bacterioruberin extracted from lab-scale cultivation of *H. salinarum* can be purchased at a price point of 553 EUR/mg (CAS: 53026-44-1) [46]. Thus, more studies on the biosynthetic pathways of carotenoids and cultivation methods are needed to obtain the maximum yield of growth and pigment production, as well as the implementation of large-scale production systems with reduced costs.

Genetic engineering offers an interesting way to enhance the biosynthesis of high-value-added products. Increasing bacterioruberin biosynthesis through the genetic manipulation of haloarchaea is now possible due to recent advances in the knowledge of carotenogenesis and the sequencing of the genomes of haloarchaea belonging to different genera. However, this approach has only been tested in some species [60]. Genetic manipulation of the carotenoid biosynthetic pathway by overexpression, edition, or knockout of specific genes of the pathway should be explored in more depth to overcome the low carotenoid concentration observed in many species.

Finally, it is worth mentioning that most studies testing the bioactive properties of bacterioruberin have been performed in vitro, so it is necessary to study whether these results can be extrapolated in vivo. Moreover, the toxicity of bacterioruberin should be thoroughly analyzed in animal and human models, and the development of efficient and stable transport systems must be achieved.

### 2.2. Bacteria

#### 2.2.1. Ecological Importance

The bacterial communities inhabiting marine environments vary from coastal to offshore areas, as in the water column, due to differences in physico-chemical and biological properties to which they are exposed. Depending on their energy and carbon sources, bacteria can be phototropic or non-phototropic.

Carotenoids are produced in all phototrophic bacteria (Table 2), and their biological function is to support photosynthesis as accessory pigments, protect the photosynthetic machinery from excess light that induces photodamage, and quench reactive oxygen species (ROS) [66,67,68]. There are four main types of bacterial phototrophs in marine microbial communities: (i) Cyanobacteria, which are mainly represented in marine environments with the genera *Synechococcus* and *Prochlorococcus* as one of the main contributors to planktonic systems [69]; (ii) anaerobic anoxygenic phototrophic bacteria such as purple sulfur bacteria, purple non-sulfur bacteria, and green sulfur bacteria, which are generally restricted to oxygen-free habitats, such as sediments and mats [67,70,71,72]; (iii) aerobic anoxygenic phototrophic bacteria, which includes members belonging to either α-, β-, or γ-proteobacteria, and inhabit the upper layer of the ocean, representing up to 15% of the total bacterial community [72]; and (iv) proteorhodopsin-containing bacteria. It is estimated that as much as half of the surface ocean bacteria could be part of them, but functional evidence is still scarce [69,72,73,74].

On the other hand, the widespread presence of carotenoids in non-phototrophic bacteria indicates their importance for their viability (Table 2). α-proteobacteria and γ-proteobacteria, together with members of the Bacteroidetes phylum, are the most abundant groups of heterotrophic bacteria in the sea [73]. Due to the absence of photosynthetic apparatus, the role of carotenoids in organoheterotrophs lies mainly in protection from photooxidative damage and in the regulation of membrane fluidity [75,76]. The production of carotenoids is proposed to be an adaptive response to certain environmental and stressor conditions, such as freeze–thaw cycles and solar radiation [77].

#### 2.2.2. Sustainability Pros and Cons

Marine bacteria could be a promising alternative for the development of sustainable processes to produce carotenoids. As with other microorganisms, the culture systems used to grow bacteria are independent of seasonal restrictions, have low utilization of space, and do not require the use of arable land. Even when more research is needed to improve bacteria as a carotenoid source, novel strategies aligned with the development of sustainable processes could be a niche for the use of marine bacteria diversity. Second- and third-generation feedstocks require the maximum utilization of resources with the generation of multiple products, as in a biorefinery, to favor the global economic balance of the process. Microorganisms are known for their ability to use a wide variety of substrates. Bacteria present certain advantages, such as a high growth rate, metabolic diversity, and easier genetic manipulation [78]. Even though up-to-date scare reports include marine bacteria, promising research is being carried out. Kristjansdottir et al. proposed that *Rhodothermus marinus* has potential for its application in biorefineries since it is a thermophile that produces various biomass-degrading enzymes and, among other interesting products, produces carotenoids [79]. Park et al. isolated *Vibrio* sp. SP1 from a starfish for its ability to grow from alginate, and after introducing genes encoding enzymes to produce lycopene and β-carotene, it could grow on brown macroalgae *Sargassum* and produce lycopene [80].

#### 2.2.3. Main Species (and Compounds) in the Context of Agro-Food and Health

Overall, the carotenoids most demanded for their importance in agro-food and health that are produced by bacteria are β-carotene, β-cryptoxanthin, zeaxanthin, astaxanthin, and canthaxanthin (Figure 1). In Table 2, a list of the species and main carotenoids produced is presented.

**Table 2:** Examples of carotenoid content in marine bacteria and cyanobacteria.

Organisms	Species	Carotenoids	Collection Sites	Experimental Conditions	Quantification/Identification Methodologies	Contents	References
Bacteria	*Rubritalea squalenifaciens* (DSM 18772^T^)	Diapolycopenedioc acid xylosylesters	Miura Peninsula, Kanagawa, Japan	Liquid culture (1.0% starch, 0.4% yeast extract, and 0.2% peptone in seawater), 30 °C, 120 rpm, 2 days	Carotenoids purification by chromatography and identification of the structures by HRESI-MS and spectroscopic analyses	2.2–10.2 mg purified from cells in a 42 L culture	[81]
	*Planococcus maritimus* strain iso-3	Methyl 5-glucosyl-5,6-dihydro-apo-4,4′-lycopenoate	Clyde estuary, UK	Marine broth 2216), 30 °C, 120 rpm, 1 day	Carotenoids purification by chromatography and identification of the structures by HRESI-MS and spectroscopic analyses	2.5 mg purified from cells in an 18 L culture	[81]
	04OKA-13-27 (MBIC08261)	(3*R*)-saproxanthin	Coast of Okinawa Prefecture, Japan	Marine Broth 2216, 30 °C, 100 rpm, 1 day	Carotenoids purification by chromatography and identification of the structures by MS, ^1^H-NMR, and CD analyses	0.3 mg purified from cells in a 2 L culture	[81]
	YM6-073 (MBIC06409)	(3*R*,2′*S*)-myxol	Coast of Okinawa Prefecture, Japan	Marine broth, 30 °C, 100 rpm, 1 day	Carotenoids purification by chromatography and identification of the structures by MS, ^1^H-NMR, and CD analyses	0.5 mg purified from cells in a 2 L culture	[81]
	*Erythrobacter rubeus* sp. nov.	Keto-spirilloxanthin	Hamdeok Beach, Jeju Island, Republic of Korea	Marine agar 2216, 25 °C	-	-	[82]
	*Flavobacterium* sp. P8	Zeaxanthin	King George Island, Antarctica	2 L medium (7 g/L peptone, 7 g/L yeast extract, and 15 g/L NaCl) in a 5 L-bioreactor, 20 °C, pH 7, 1 vvm airflow, 20% pO_2_, 72 h	HPLC	2.15 ± 0.15 mg/L, DW	[83]
	*Brevundimonas scallop*	Astaxanthin, 2,2′-dihydroxy-astaxanthin, and 2-hydroxy-astaxanthin	Nanao Island of Guangdong Province, China	100 mL LB medium, 25 °C, 150 rpm, 3 days	Spectrophotometry	104.29 ± 4.98 µg/g astaxanthin, 1068.46 ± 52.42 µg/g 2,2′-dihydroxy-astaxanthin, and 130.36 ± 6.11 µg/g 2-hydroxy-astaxanthin, DW	[84]
	*Erythrobacter citreus* LAMA 915	Adonixanthin, caloxanthin, canthaxanthin, erythroxanthin sulfate, nostoxanthin, and zeaxanthin	Atlantic Ocean (30° S, 36° W)	Soluble fish hydrolysate 0.5%	Spectrophotometry	1.5 mg/L total carotenoids, FW	[85]
	*Erythrobacter flavus* KJ5	Caloxanthin, caloxanthin sulfate, caloxanthin sulfate isomer, β-carotene, β-carotene *cis* isomer, β-cryptoxanthin, nostoxanthin sulfate, nostoxanthin isomer, zeaxanthin, zeaxanthin isomer, zeaxanthin sulfate, and zeaxanthin sulfate *cis* isomer	Hard coral Acropora nasuta, Karimunjawa Islands, Indonesia	Shioi broth, 28 °C, under dark conditions, 48 h	-	-	[86]
	*Planococcus* sp. Eg-Natrun	Astaxanthin and β-carotene	Umm Risha Lake, Wadi El-Natrun, south of Al-Buhayrah province, Egypt	E1 rich marine medium (C/N~3.95), 37 °C, 150 rpm, 3 days	Spectrophotometry	1024 ± 53 µg/g, DW	[87]
	*Brevundimonas* sp. strain N-5	Astaxanthin	Shimoda Port, Shizuoka Prefecture on the Pacific coast of Middle Japan	NB broth, 150 rpm, 2 days	HPLC	601.2 μg/g total carotenoids, 364.6 μg/g astaxanthin (3*S*, 3′*S*), DW	[88]
	*Paracoccus* sp. strain N-81106	Astaxanthin	Iwate, Japan	OEG medium, 25 °C, 150 rpm, 18 h	HPLC	0.9 ± 0.1 mg/L astaxanthin,3.1 ± 0.2 mg/L total carotenoids, FW	[89]
	*Vitellibacter* sp. BW	Zeaxanthin and β-carotene	East and West coast of India (Tuticorin, Mandappam, Rameshwaram, and Mangalore)	Marine broth, 37 °C, 200 rpm, 5 days	HPLC	115.7 ± 5.0 mg/g total carotenoid, FW	[90]
	*Arenibacter* sp. 4W	Zeaxanthin and β-carotene	East and West coast of India (Tuticorin, Mandappam, Rameshwaram, and Mangalore)	Marine broth, 37 °C, 200 rpm, 5 days	HPLC	99.3 ± 21.9 mg/g total carotenoid, FW	[90]
	*Paracoccus* sp. LL1 (KP288668)	Astaxanthin	Lonar lake, India	Fed-batch culture in minimal medium supplemented with 20 g/L of glucose. Fermenter with a membrane of cell filtration, 1.5 vvm airflow, 300 rpm, DO > 20%	HPLC	8.51 ± 0.20 mg/L intracellular astaxanthin, 10.2 ± 0.24 mg/L extracellular astaxanthin, FW	[91]
	*Paracoccus haeundaensis* KCCM 10460	Astaxanthin	-	Co-culture with lactic acid bacteria. PMF medium, 25 °C, 160 rpm, 1 vvm airflow, 72 h	HPLC	821.09 ± 30.98 µg/g, DW	[92]
	*Paracoccus zeaxanthinifaciens* ATCC 21588	Zeaxanthin	Purchased from BCCM/LMG, Ghent University, Belgium	Culture media, 30 °C, 180 rpm, 72 h	TLC and spectrophotometry	11.63 mg/L, FW	[93]
	*Paracoccus zeaxanthinifaciens* ATCC 21588	Zeaxanthin	Purchased from BCCM/LMG, Ghent University, Belgium	Culture media in a bubble column reactor, 30 °C, 80 h	HPLC	13.76 ± 0.14 mg/L, FW	[94]
Cyanobacteria	*Anabaena* sp. PCC 7120	Canthaxanthin, β-carotene, echinenone, ketomyxol 2′-fucoside, and myxol 2′-fucoside	A gift from Waseda University, Japan	BG-11 medium, 26–28 °C, 110 rpm, white fluorescent light (30–40 µE m^−2^ s^−1^), 2 weeks	HPLC	Relative molecular masses (%): 1 canthaxanthin, 62 β-carotene, 25 echinenone, 4 ketomyxol 2′-fucoside, and 8 myxol 2′-fucoside	[95]
	*Anabaena variabilis* IAM M-3 (=PCC 7118, ATCC 27893)	Canthaxanthin, β-carotene, echinenone, ketomyxol 2′-fucoside, 3′-hydroxyechinenone, and myxol 2′-fucoside	A gift from Waseda University, Japan	BG-11 medium, 26–28 °C, 110 rpm, white fluorescent light (30–40 µE m^−2^ s^−1^), 2 weeks	HPLC	Relative molecular masses (%): 4 canthaxanthin, 38 β-carotene, 33 echinenone, 13 ketomyxol 2′-fucoside, 1 3′-hydroxyechinenone, and 11 myxol 2′-fucoside	[95]
	*Nostoc punctiforme* PCC 73102 (=ATCC 29133)	Canthaxanthin, β-carotene, echinenone, ketomyxol 2′-fucoside, and myxol 2′-fucoside	A gift from Kanazawa University, Japan	BG-11 medium, 26-28 °C, 110 rpm, white fluorescent light (30–40 µE m^−2^ s^−1^), 2 weeks	HPLC	Relative molecular masses (%): 13 canthaxanthin, 45 β-carotene, 17 echinenone, 13 ketomyxol 2′-fucoside, and 11 myxol 2′-fucoside	[95]
	*Anabaena variabilis* ATCC 29413 (=IAM M204)	Canthaxanthin, β-carotene, echinenone, 4-hydroxymyxol, and myxol	A gift from Waseda University, Japan	BG-11 medium, 26–28 °C, 110 rpm, white fluorescent light (30–40 µE m^−2^ s^−1^), 2 weeks	HPLC	Relative molecular masses (%): 22 canthaxanthin, 51 β-carotene, 20 echinenone, 2 4-hydroxymyxol, and 5 myxol	[96]
	*Synechocystis* sp. PCC 6803	β-carotene, deoxymyxol 2′-dimethyl-fucoside, echinenone, 3′-hydroxyechinenone, myxol 2′-dimethyl-fucoside, and zeaxanthin	A gift from Waseda University, Japan	BG-11 medium supplemented with 10 mM HEPES buffer (pH 7.5) in 10 L carboys, 30 °C with aeration and irradiation (40–100 E/m^2^·s)	HPLC	Relative molecular masses (%): 26 β-carotene, 1 deoxymyxol 2′-dimethyl-fucoside, 18 echinenone, 4 3′-hydroxyechinenone, 36 myxol 2′-dimethyl-fucoside, and 14 zeaxanthin	[97]

CD, circular dichroism; DW, dry weight; FW, fresh weight; ^1^H-NMR, proton nuclear magnetic resonance; HPLC, high-performance liquid chromatography; HRESI-MS, high-resolution electrospray ionization-mass spectroscopy; MS, mass spectrometry; TLC, thin-layer chromatography.

Among Bacteroidetes, Flavobacteriaceae is an important family with several species that have been reported to produce carotenoids. Marine *Flavobacterium*, *Chryseobacterim*, *Formosa*, *Vitellibacter*, and *Arenibacter* are genera with species known for producing zeaxanthin, β-cryptoxanthin, and β-carotene [98,99,100].

Regarding Alphaproteobacteria, the genera *Paracoccus* and *Brevindimonas* have been widely studied for their capacity to produce carotenoids. *Paracoccus haeundaensis* sp. and *Paracoccus* sp. LL1 (KP288668) were reported to produce astaxanthin [91,101], while *Paracoccus zeaxanthinifaciens* produces mainly zeaxanthin [93,102]. *Brevundimonas scallop* and *Brevundimonas* sp. N-5 produce astaxanthin [84,88].

Regarding photosynthetic bacteria, several members are reported as carotenoid producers. Members of *Anabaena*, *A. variabilis* ATCC 29413, *A. variabilis* IAM M-3, and *Anabaena* sp. PCC 7120, were reported for their ability to produce ꞵ-carotene, echinenone, and canthaxanthin (Figure 1) [103]. *Synechococcus* species were studied for the production of carotenoids: zeaxanthin, cryptoxanthin, myxoxanthophyll (myxol-2′-fucoside), echinenone, 3′-hydroxyechinenone, and synechoxanthin. *Synechococcus elongatus* PCC 7942 is a cyanobacterium that synthesizes zeaxanthin as one of the predominant carotenoids [104]. Other carotenoids-producing cyanobacteria are *Nostoc punctiforme* PCC 73102, which produces β-carotene, echinenone, and canthaxanthin; *Thermosynechococcus elongatus* BP-1 produces mainly β-carotene and nostoxanthin; *Gloeobacter violaceus* PCC 7421 produces β-carotene and echinenone; and *Prochlorococcus marinus* MED4 produces β-carotene, β-cryptoxanthin, and zeaxanthin [103].

#### 2.2.4. Production Approaches and Production Data in Different Continents

The industrial production of bacterial carotenoids is still in development. The literature reports laboratory-scale experiments and scales up to laboratory bioreactors operated as batch cultures, with alternative approaches such as fed-batch and co-culture. Most of the research refers to aquatic bacteria, but not those of marine origin. Joshi et al. studied marine *Paracoccus zeaxanthinifaciens* as an interesting strain for zeaxanthin production [93]. These authors developed a media culture using surface response methodology and an artificial neural network that allowed them to reach an overall yield of 11.6 mg/L after 72 h of culture. Vila et al. [83] formulated a media culture to produce zeaxanthin by a marine Antarctic *Flavobacterium* sp. P8 utilizing factorial designs and later used a 5 L bioreactor in batch mode to obtain 2.15 mg/L of zeaxanthin. A strategy of co-culture was studied by Choi et al. [92] in the production of astaxanthin by *Paracoccus haeundaensis* sp. This strategy, coupled with the optimization of the media culture composition, led to an increase in astaxanthin production of 2.5 times compared to *P. haeundaensis* cultivation in Luria–Bertani broth medium. Genetic engineering has been applied to improve carotenoids yields in the last few decades. Sarnaik et al. [104] developed a genetically modified strain PCC 7942 (*Synechococcus* 79R48) by homologous recombination, which improves β-carotene flux toward zeaxanthin synthesis. The modified strain improved zeaxanthin production two times relative to the wild-type strain. In addition, heterologous production of astaxanthin from marine bacterial genes was achieved in *Synechocystis* sp. PCC 6803 [105].

#### 2.2.5. Current and Prospective Applications

Nowadays, the only bacteria commercially exploited as a carotenoid source is *Paracoccus carotinifaciens*, which has approvals as a feed product from EFSA and as an astaxanthin-rich extract to be used in food from the FDA. Although it is not of marine origin, it shows that bacteria are a viable option for carotenoid production if they fulfill scale-up requirements and economic balance. To the authors’ knowledge, there are not yet any products for food or health purposes on the market. Resistance to bacteria-based products has been presented as a possible cultural obstacle to overcome [78].

Part of the ongoing research involves modifications of the carotenoid biosynthetic pathways of the well-known *Corynebacterium glutamicum* and heterologous expression in *E. coli* to increase carotenoid production [106,107,108]. Besides, the application of genetic manipulation tools to increase the carotenoid yields of marine carotenogenic strains is still promising [89]. Then, marine bacteria could be a cell factory [109], a source of carotenogenic genes contributing to the improvement of tools for heterologous expression, as well as a source of novel carotenoids with potential new applications [110]. On the other hand, the inclusion of carotenoids as a high-value product in the development of biorefineries in the context of sustainable processes could be promising. The diversity of marine bacteria may contribute to providing appropriate degrading properties of different feedstocks combined with the production of carotenoids.

#### 2.2.6. Research Needs

The existence of much more diversity than that recovered by classical culture techniques from marine environments has challenged bioprospecting strategies, and new approaches incorporating heterologous expressions of metagenomic data are possible but difficult [111]. It is still necessary to isolate and culture bacteria to study the production of bioactive metabolites such as carotenoids [112], and meanwhile, improvements in culture methods are reported [113,114,115,116,117,118].

Much of the chemical identification of new carotenoids is carried out by mass spectrometry analysis, but the lack of commercial standards (for identification and quantification) and chemical data literature slows progress [35]. Besides, among the wide variety of carotenoids produced by bacteria, only a few have proven applications. New and rare carotenoids are frequently characterized by antioxidant activity and preliminary assays with cell lines, but more research is required to evaluate their real potential application [81].

Finally, there is a lack of economic, technological, and environmental assessment research to guide the progress of the bioprocess of bacterial carotenoid production. As mentioned above, integrating the carotenoid production process into a multi-product biorefinery approach could improve competition with the actual production processes.

### 2.3. Macroalgae

#### 2.3.1. Ecological Importance

Macroalgae play an important role in coastal ecosystems, mainly as a habitat and food source [119,120]. They require certain salinity levels, sunlight, and adequate attachment substrates, although they can also float. Macroalgae are at the basis of the food chain, providing vegetal material and nutrition to other marine organisms, partly through associated microbiota [121]. They form complex structures that serve as an important shelter, support, or breeding substrate. Similarly, macroalgae vegetation is essential for the early developmental stages of many commercially interesting fish species [122,123]. Large seaweed can even contribute to protecting the shoreline from climate-imposed changes [124,125]. Due to their large surface area, macroalgae filter surrounding water, retain excessive nutrients, and mitigate pollution for the integrity of their marine environment [126]. Macroalgae can also act as carbon sinks through the uptake of atmospheric carbon dioxide and the export of dissolved organic carbon to offshore areas [7,127]. In addition, seaweeds contribute to oxygen production during the daytime due to their photosynthetic activity. Overall, macroalgae are regarded as essential in the aquatic trophic chain web, and some of the environments to which they belong are regarded as among the most productive habitats on the planet. Other ecosystem services identified are their use as ingredients in the formulation of feeds, foods, fertilizers, drugs, and other products for human consumption [5].

The photosynthetic pigments and their combinations present in seaweeds have high taxonomic importance in their classification. Green algae are a large group, of which one class is Chlorophyceae. Chlorophyceae are the closest to terrestrial green plants and contain chlorophylls a and b and carotenoids. The Phaeophyceae, which represent one class of brown algae, contain mainly chlorophyll c and the brown carotenoid pigment fucoxanthin. Rhodophyceae, or red seaweed, is a group distinguished by the presence of pigments such as chlorophyll, carotenoids, and the phycobiliprotein phycoerythrin. Carotenoids in seaweed can function as light energy harvesters (passing on light excitation to chlorophyll) and antioxidants that inactivate the ROS formed under exposure to high light and air [128,129].

#### 2.3.2. Sustainability Pros and Cons

Concepts including sustainability and the circular economy are a prerequisite that is now associated with food production. The agro-food system is always in search of sustainable and healthy diets for a growing population [130,131]. Today, most carotenoids are chemically synthesized or extracted from land plants. Therefore, macroalgae could serve as an alternative source of carotenoids, with the prerequisite that algae production and carotenoid extraction can be sustainable. This is well represented in the literature, with a focus on induced carotenoid synthesis in green [132] or red algae [133]. Several extraction techniques have been investigated and are suggested to be faster, more sustainable, and more efficient than traditional methods to isolate bioactive compounds from algae [134]. Finally, the sustainability of seaweed farming is also yet to be demonstrated for its production in Europe [135,136].

#### 2.3.3. Main Species (and Compounds) in the Context of Agro-Food and Health

The main bioactive compounds in macroalgae are polysaccharides, phenolic compounds, carotenoids, vitamins, minerals, and peptides [137,138]. Carotenoids are photosynthetic and bioactive compounds found in all of the seaweed divisions (Table 3), playing critical roles in photosynthesis. Seaweed carotenoids are generally localized in the chloroplast or accumulated in vesicles, the cytoplasmic matrix, or bound to membranes and other macromolecules in the intracellular space. Carotenoids in seaweeds are synthesized from pyruvate and/or acetyl-CoA. The first carotenoid in the isoprenoid route is phytoene, from which lycopene is formed after several desaturation steps. Lycopene is further transformed sequentially into compounds including α-carotene and lutein in red and green algae or β-carotene and zeaxanthin in the three types of macroalgae. Green algae further transform zeaxanthin into violaxanthin and neoxanthin, while only brown algae finally form fucoxanthin [139,140,141]. Furthermore, different carotenoids are synthesized in response to exposure to light and air since they scavenge ROS with their photoprotective properties [139,142].

In the marine environment, carotenoids are widespread in seaweeds (Table 3). Green seaweeds have a carotenoid profile very similar to that of green vegetables (with lutein and β-carotene as major carotenoids) along with violaxanthin, neoxanthin, and others [35,132]. In addition to fucoxanthin, Phaeophyceae are rich sources of β-carotene and violaxanthin and also contain ε-carotene, antheraxanthin, diatoxanthin, diadinoxanthin, and neoxanthin at lower concentrations. α-carotene, β-carotene, zeaxanthin, and lutein are commonly found in Rhodophyceae [143].

One very well-studied carotenoid in seaweeds is fucoxanthin, the brown pigment that colors brown seaweeds and, in particular, kelps, as well as diatoms. Fucoxanthin is one of the most abundant carotenoids, contributing around 10% of the estimated total production of carotenoids in nature [144]. While fucoxanthin from unicellular diatoms is interesting for industrial production, brown seaweed is the only dietary source of fucoxanthin. Fucoxanthin is produced industrially using macroalgae, such as *Laminaria japonica* or *Undaria pinnatifida*, although its fucoxanthin content is low (0.2–0.6% of dry weight) [145]. A survey in Japan showed that while the intake of carotenes and other xanthophylls was covered by different food sources, fucoxanthin was mainly provided by the intake of the brown seaweed wakame [146]. The fucoxanthin content will vary between different species and genera and according to environmental factors, such as geographical location and season. Furthermore, the detected content will also be influenced by the extraction method [139].

**Table 3:** Examples of carotenoid content in marine macroalgae.

Organisms	Species	Carotenoids	Collection Sites	Experimental Conditions	Quantification/Identification Methodologies	Contents	References
Macroalga (brown)	*Sargassum horneri*	Fucoxanthin	Nesaki, Hokkaido (41°45′ N, 140°49′ E) and Matsushima, Miyagi (38°23′ N, 141°04′ E), northern seashore of Japan	Mariculture of collected thalli in Usujiri, Japan	HPLC	1.35–4.5 mg/g DW	[147]
	*Cystoseira hakodatensis*	Fucoxanthin	Usujiri, Hokkaido (41°56′ N, 140°57′ E), Japan	Natural growth in Usujiri, Japan	HPLC	0.6–4.1 mg/g DW	[147]
	15 species of brown seaweed	Fucoxanthin	Wild harvest in intertidal zones in Shinori and Nesaki, Japan	Natural growth	HPLC	0.1–3.7 mg/g DW	[148]
Macroalga (green)	*Ulva* spp.	Total carotenoids	Wild harvest in Inter- and subtidal zones in India, Egypt, China, Spain, and Chile	Natural growth	Spectrophotometry	1.25–4.6 mg/g DW	[132]
	*Ulva compressa*	Lutein, neoxanthin, violaxanthin, and zeaxanthin	Coasts of Mangaluru (12°45′31.7″ N 74°51′53.2″ E to 13°06′25.9″ N 74°46′ 03.6″ E), India	Natural growth	HPLC	4.7 μg/g lutein, 3.8 μg/g neoxanthin, 4.0 μg/g violaxanthin, and 3.9 μg/g zeaxanthin, DW	[149] *
	*Chaetomorpha antennia*	Lutein, neoxanthin, violaxanthin, and zeaxanthin	Coasts of Mangaluru (12°45′31.7″ N 74°51′53.2″ E to 13°06′25.9″ N 74°46′ 03.6″ E), India	Natural growth	HPLC	141.3 μg/g lutein, 33.3 μg/g neoxanthin, 33.7 μg/g violaxanthin, and 34.6 μg/g zeaxanthin, DW	[149]
Macroalga (red)	*Grateloupia* sp.	Lutein and zeaxanthin	Coasts of Mangaluru (12°45′31.7″ N 74°51′53.2″ E to 13°06′25.9″ N 74°46′ 03.6″ E), India	Natural growth	HPLC	166.6 μg/g lutein and 36.3 μg/g zeaxanthin, DW	[149]
	*Pyropia yezoensis*	α-carotene, β-carotene, lutein, zeaxanthin	Thalli of *P. yezoensis* strain U-51, maricultured at Shichigahama, Miyagi, Japan	Mariculture	HPLC	0.7 mg/g α-carotene, 1.8 mg/g β-carotene, 1.4 mg/g lutein, and 0.15 mg/g zeaxanthin, DW	[150]

DW, dry weight; HPLC, high-performance liquid chromatography. * In this reference, more species of algae have been analyzed, in addition to those indicated in the table.

#### 2.3.4. Production Approaches and Production Data in Different Continents

Seaweed accounts for approximately 50% of the production of marine biomass by aquaculture, which is the fastest-growing component of food production with an estimated growth of >7%/year, ~3–5-fold that of agriculture (2%/year), livestock (2.6%/year), and wild fisheries (0.1%/year) [151].

Almost all the production happened in Asian countries (especially China and Indonesia). To be sustainable, the activities associated with seaweed farming require coordinated and participatory governance, regulations, and best practices. Globally, seaweed mariculture is still marginal in Europe although is expected to grow dramatically to become important in the European Blue Growth and Bioeconomy strategies [152,153,154]. The main exploited algae species in Europe are *Laminaria hyperborea*, *Laminaria digitata*, and *Ascophyllum nodosum*, which, as kelp forests, are considered among the world’s most ecologically dynamic and biologically diverse habitats [155,156]. Other seaweeds such as *Alaria esculenta*, *Pyropia yezoensis*, and *Palmaria palmata* are ingredients in many European cuisines [10].

#### 2.3.5. Current and Prospective Applications

There is an important interest in bioactive molecules with antioxidant and anti-inflammatory effects, which makes seaweed compounds interesting ingredients for functional food and cosmetic formulations. Fucoxanthin is the most studied carotenoid in seaweed and has exhibited antioxidant and anti-inflammatory effects, which are extensively reviewed [139,157,158,159,160]. Olsthoorn et al. [160] have recently published a comprehensive review in which they identify four inflammatory principles on which brown seaweeds have an effect. Fucoxanthin is effective in two of them, namely regulating ROS and innate immune responses, thus reducing pro-inflammatory cytokines induced by both [160].

Only two studies were found on clinicaltrial.gov when searching for “fucoxanthin NOT microalgae”, which plan to investigate the effect of fucoxanthin supplementation in non-fatty liver disease and metabolic syndrome.

In animal studies, the administration of fucoxanthin-rich algae extracts increased antioxidative enzymes and decreased ROS in two mouse models of diabetes and colitis, respectively, and in cisplatin-induced hamsters [161,162,163].

Fucoxanthin treatment ameliorated histological damage and inflammatory levels of PGE_2_ in the colon of mice after induced colitis and downregulated the inflammation-related enzyme cyclooxygenase-2 (COX-2) and the transcription factor nuclear factor kappa B (NF-kB) [164]. Oral and percutaneous administration of fucoxanthin reduced ear swelling in mice and limited the induction and activity of COX-2, phospholipase A2 (PLA_2_), and hyaluronidase (HA) [165]. Fucoxanthin administration also reduced inflammatory cytokines (IL-1β, IL-6, and TNF-α) in an obesity mouse model [166] and by lipopolysaccharide (LPS)-induction as well as depression-associated behavior in the LPS-stimulated mice [167]. These effects have also been reviewed in light of their potential protective role in several connected pathologies, such as liver and skin damage or cardiovascular and metabolic disease [159]. Miyashita and Hosokawa have also reviewed the potential uses of fucoxanthin in the management of obesity and diabetes, including a downregulation of inflammatory adipokines [158,168] and reduced body weight and abdominal fat in overweight Japanese subjects [169].

Yang et al. summarize the studies in cell models and mice, which show the potential of fucoxanthin for therapeutic efficacy in neurodegenerative disorders [164]. Of special interest, fucoxanthin had a neuroprotective effect against cerebral ischemic/reperfusion injury through activation of the antioxidative response pathway involving nuclear factor erythroid 2-like 2 (NFE2L2/Nrf2) and heme oxygenase 1 (HMOX-1) [170]. Fucoxanthin also reduced neuroinflammation in a mouse model of Parkinson’s disease [171].

Here, we only mention some in vivo studies illustrating the anti-inflammatory and antioxidative effects of fucoxanthin, as we understand these to be most relevant for its final application. An extensive body of work in vitro has been published to evaluate this and other seaweed-derived carotenoids, with some described in Section 5 [139,172,173]. There are a few additional studies with whole seaweed and mixed extracts, which also provide valuable information on the effects of dietary intake of seaweed [174,175]. During uptake, fucoxanthin is metabolized to fucoxanthinol and amarouciaxanthin A, which were found in the adipose tissue, kidney, heart, lung, and spleen of mice [176,177]. In humans, fucoxanthin intake led to a peak of fucoxanthinol in plasma of 44.2 ± 14.9 nM after 4 h, gradually decreasing within 24 h [178,179]. Therefore, the bioavailability of fucoxanthin is lower and more transient than that of other dietary carotenoids such as β-carotene and lutein [178,180]. This underlines the importance of in vivo studies that take into account the limiting steps of metabolism and uptake.

#### 2.3.6. Research Needs

The anti-inflammatory and antioxidant effects are among the three most commonly published pharmacological properties of fucoxanthin in recent years. However, most studies are based on cell and mouse models, with only a minority focusing on clinical interventions [181]. Thus, more clinical studies are needed to confirm and fully exploit the described anti-inflammatory and antioxidant effects of this carotenoid. Compared to fucoxanthin, there is almost no literature on the potential health effects of other carotenoids derived from green or red seaweeds, including their metabolized or degradation products, which could also have beneficial effects. For example, a carotenoid degradation product, 3-hydroxy-4,7-megastigmadien-9-one, extracted from the green seaweed *Ulva pertusa*, was shown to limit the inflammatory response to microbial products in mouse innate immune cells [173].

In light of increasing interest in carotenoids from alternative sources, the production of seaweeds with a higher content of carotenoids could be one approach. Aquaculture technology would need to be optimized with a better understanding of how environmental parameters affect seaweed physiology and therefore their carotenoids content.

Finally, the economic and environmental sustainability as well as the safety of the whole value chain from seaweed to the carotenoid final product still need to be validated and documented according to the regulatory framework to ensure sustainable and safe use of this ingredient.

### 2.4. Microalgae

#### 2.4.1. Ecological Importance

Microalgae are a heterogeneous group of photosynthetic microorganisms of high ecological importance. They are responsible for approximately half of the global CO_2_ fixation, which makes them “cell factories” capable of taking up the main greenhouse gas in the atmosphere [182]. Their cultivation does not require arable land, and microalgal biomass can be produced (between 40 and 100 t/ha year of dry weight) 10–50 times faster than higher plants [183]. Additionally, most microalgae show high robustness when exposed to different types of abiotic stress, such as extreme temperatures, nutrient depletion, high salinity, or the presence of pollutants. These characteristics make it possible for microalgae to inhabit almost all ecosystems on Earth, demonstrating an enormous range of ecological adaptation capacities [184].

In addition, microalgae are at the base of the marine food chain, being the most abundant primary producers in aquatic ecosystems [185]. They are widely used as a nutritional supplement in aquaculture due to their high nutritional qualities and their potential to produce a wide range of high-value compounds, such as polyunsaturated fatty acids (PUFAs), sterols, vitamins, polyphenols, or carotenoids [186]. Some of these compounds are also synthesized for the pharmaceutical or cosmetic industry and marked as food supplements.

#### 2.4.2. Sustainability Pros and Cons

There is an increasing interest in addressing the integral utilization of all microalgal components, following a biorefinery approach, and in integrating their production with the utilization of residual waste waters or the capture of CO_2_ through a circular economy perspective to reduce the costs of microalgal biomass culturing and harvesting [187,188]. These studies are mainly focused on the removal of pollutants and the production of low-value compounds, such as biofuel, bioethanol, or animal feed. An example of this is the cultivation of *Chlorella vulgaris* and *Chlorella sorokiniana* in wastewater from milk processing, dairy wastewater, or wastewater from the wine industry that obtains large amounts of lipids, mainly palmitic, oleic, and linoleic acids [189,190,191]. *Arthrospira platensis* (Spirulina) was also grown in an anaerobic digestion effluent containing Cu^2+^ and Zn^2+^, and the obtained biomass was used as feed for animals [192]. Moreover, there are some studies that describe the culture of the microalgae *Dunaliella* sp. and *Haematococcus pluvialis* in digested poultry litter or treated primary wastewater for the production of β-carotene and astaxanthin, respectively. These microalgal species are the first natural source of these highly demanded carotenoids, and their culture in wastewater can improve their feasibility [193,194].

#### 2.4.3. Main Species (and Compounds) in the Context of Agro-Food and Health

Carotenoids are among the high-value compounds produced from microalgae, with several applications and high commercial interest. Some carotenoids, such as β-carotene, astaxanthin, lutein, or fucoxanthin, obtained from different microalgae are widely commercialized for cosmetic and pharmaceutical applications [186]. Although many species of microalgae are able to produce these compounds, only a few of them synthesize enough amounts of carotenoids to make industrial processes economically feasible (Table 4).

*Dunaliella salina* is one of the main producers of β-carotene and is considered the best commercial source of this naturally produced compound. This green microalga can accumulate up to 13% of its total biomass in β-carotene, making it a much better producer than other species such as *Chromochloris zofingiensis* or *Arthrospira platensis* (Spirulina), which yield up to 1–2% of their dry biomass [145]. The β-carotene synthesized by *Dunaliella salina* has exhibited health-promoting actions in experiments of different nature, such as protective effects against atherosclerosis, protection against UV-induced erythema, and oxidative damage in humans [195].

Another microalga able to produce high amounts of carotenoids in industrial processes is *Haematococcus pluvialis*. This microalga belongs, as *Dunaliella salina*, to the chlorophyte phylum and is the main producer of astaxanthin, a keto-carotenoid with high antioxidant capacity in vitro. *Haematococcus pluvialis* can produce up to 4–5% of its dry biomass as astaxanthin, obtaining higher yields than other strains capable of producing this compound, such as *Chromochloris zofingiensis* or *Chlorococcum* sp. [196]. Natural astaxanthin produced by *Haematococcus pluvialis* has previously been reported to have 10 times the in vitro antioxidative capacity of other carotenoids, such as zeaxanthin, lutein, canthaxanthin, or β-carotene, as well as anti-aging, anti-inflammatory, and anti-atherosclerotic properties [196].

Other green microalgae belonging to the *Chlorella*, *Muriellopsis*, or *Scenedesmus* genera are able to produce considerable amounts of lutein. However, its lutein content (0.5–1.2% of dry biomass) is much lower than the β-carotene or astaxanthin content in *Dunaliella* or *Haematococcus*, respectively [197]. This low productivity has restricted the industrial production of lutein from microalgae, whose main industrial source continues to be the petals of higher plants, such as marigolds [145]. However, microalgae can produce lutein in its free form, while in marigolds, lutein is mainly esterified with fatty acids [145]. One of the most promising species for the production of lutein is *Chlorella vulgaris*. This microalga can accumulate around 0.8% of the total dry weight in lutein and is one of the microalgae species allowed for human consumption [198,199]. However, further studies are necessary for the industrial production of *Chlorella vulgaris* as a producer of this health-promoting carotenoid [195].

Some marine microalgal species are also able to produce carotenoids. Diatoms, including *Phaedoactylum tricornutum*, *Cylindroteca closterium*, *Nitzschia laevis*, and *Odontella aurita*, among others, can produce outstanding amounts of fucoxanthin, up to 2.6% of dry biomass [200].

**Table 4:** Examples of carotenoid content in marine microalgae.

Species	Carotenoids	Collection Sites	Experimental Conditions	Quantification/Identification Methodologies	Contents	References
*Chlorella vulgaris* 211/52	β-carotene, lutein	Grüental campus of the Zurich University of Applied Sciences, Wädenswil, Switzerland	Open thin-layer bioreactor	HPLC	50 μg/g β-carotene, 90 μg/g lutein, DW	[201]
*Dunaliella* sp. FACHB-558	β-carotene	Institute of Hydrobiology, Chinese Academy of Sciences, China	Two-step cultivation in anaerobically digested poultry litter wastewater	Spectrophotometry	7.26 mg/L, FW	[193]
*Isochrysis zhangjiangensis*	Fucoxanthin	Chinese Academy of Sciences, China	Photo-autotrophically cultured at 23 °C in a bubble column photobioreactors, supplemented with f/2 medium (nutrient, trace metal, and vitamin solutions)	HPLC	23 μg/g, DW	[202]
*Haematococcus plivualis*	Astaxanthin	Umeå University, Sweden	First step: inoculum in Bold’s basal medium, OD_750_ of 0.1, 20 °C, cool white LED, 1 L/min of air, 7 days. Second step: grown in a multi-cultivator with cool white LED	HPLC	19.1 mg/g, DW	[203]
*Desmodesmus* sp.	Lutein and zeaxanthin	Isolated from a wastewater treatment system, Kalundborg Kommune, Copenhagen, Denmark	Industrial wastewater in Schott bottles, stirred, aerated (2% CO_2_), and with fluorescent lights (200 µmol photon/m^2^·s)	HPLC	5.11 mg/g lutein and 0.28 mg/g zeaxanthin, DW	[204]
*Nannochloropsis salina* 40.85	β-carotene and violaxanthin	Algae culture collection (SAG), University of Gottingen, Germany	Industrial wastewater in Schott bottles, stirred, aerated (2% CO_2_), and with fluorescent lights (200 µmol photon/m^2^·s)	HPLC	2.22 mg/g β-carotene and 1.68 mg/g violaxanthin, DW	[204]
*Phaeodactylum tricornutum*	Diadinoxanthin and diatoxanthin	-	Industrial wastewater in Schott bottles, stirred, aerated (2% CO_2_), and with fluorescent lights (300 µmol photon/m^2^·s)	HPLC	2.17 μg/g diadinoxanthin and 1.55 μg/g diatoxanthin, DW	[204]
*Chlorella* C.S1	β-carotene and lutein	-	Industrial wastewater in flat panel reactors with fluorescent lights (2000 µmol photon/m^2^·s)	HPLC	1.4 mg/g β-carotene and 3.22 mg/g lutein, DW	[204]
*Nannochloropsis limnetica* 18.99	Neoxanthin and violaxanthin	Algal culture collection (SAG), University of Gottingen, Germany	Industrial wastewater in Schott bottles, stirred, aerated (2% CO_2_), and with fluorescent lights (200 µmol photon/m^2^·s)	HPLC	0.42 mg/g neoxanthin and 1.22 mg/g violaxanthin, DW	[204]
*Chlorococcum* sp.	Lutein	Umeå University, Sweden	Multi-cultivator with high light/cold stress in BG11	HPLC	15.5 mg/g, DW	[205]
*Scenedesmus* sp.	Lutein	Umeå University, Sweden	Multi-cultivator with high light/cold stress in BG11	HPLC	10.7 mg/g, DW	[205]

DW, dry weight; HPLC, high-performance liquid chromatography.

#### 2.4.4. Production Approaches and Production Data in Different Continents

The industrial production of microalgae throughout the world is under development. The microalgal global market was estimated at between USD 3.4 and USD 3.9 billion in the period 2018–2020, and it is expected to increase to USD 4.6–5.1 billion by 2027 [206,207]. Although the values of global markets will reach USD 800 million for astaxanthin, USD 620 million for β-carotene, or USD 358 million for lutein by the end of 2026 [145], around 95% of the industrial production of carotenoids is performed by chemical synthesis due to its lower cost.

For astaxanthin, while synthetic production is about USD 1000–2000 per kg; production from *Haematococcus* (around 1% of total production) has a cost between USD 1800 and 7000 per kg, depending on the location [208]. However, naturally produced astaxanthin is thought to have a stronger antioxidant capacity, being over 50 times stronger in singlet oxygen quenching and 20 times stronger in free radical elimination [209]. Natural astaxanthin is mainly obtained from *Haematococcus pluvialis.* This microalga can have two different cell morphologies: a green vegetative form and a red cyst form. Astaxanthin production in *H. pluvialis* is usually performed based on these two different morphologies, with a first stage focusing on developing their green form, which includes the production of biomass, followed by a second red stage where the astaxanthin is produced [145,195]. The production of natural astaxanthin from *H. pluvialis* is mainly concentrated in the USA, Europe, and Asia-Pacific countries, which are the main manufacturers of the companies Algatechnologies Ltd. in Israel, BluebioTech International GmbH in Germany, Algalif in Iceland, Algae Health Sciences and Cyanotech Corporation in the USA, AstaReal AB in Japan, BGG (Beijing Ginko Group) in China, and Parry’s Pharmaceuticals in India [208].

In the case of β-carotene, which is the other pigment produced by microalgae on an industrial scale, the cost of its production from *Dunaliella salina* is still higher than the chemically synthesized one. Nevertheless, synthetic β-carotene is composed only of the all-*trans* isomer, whereas natural β-carotene, as found in this microalga, is a mixture of the all-*trans* and 9-*cis* isomers, which may exhibit more beneficial properties [210]. The main strategies to increase the production of β-carotene in *D. salina* are to subject them to salt stress, high light, and N depletion. However, these stress conditions can reduce the production of biomass and, as a consequence, the yield of this carotenoid. Different solutions have been exploited to balance biomass and β-carotene production being the most common of the two-stage processes [211,212]. In these systems, there is a first stage focused on biomass production and a second one that improves the production of β-carotene. The natural production of β-carotene from *D. salina* is mainly found in Asia-Pacific countries, followed by Europe and Australia. The main manufacturers of this compound are Seagrass Tech Private Limited in India, Hangzhou OuQi Food Co. and Shaanxi Rebecca Bio-Tech Co. in China, Algalimento SL and Monzón Biotech SL in Spain, IBR Ltd. in Israel, and Plankton Australia Pty Ltd. in Australia.

The industrial production of fucoxanthin by the above-mentioned diatoms (Section 2.4.3) could be a more economically feasible approach if combined with the co-production of other high-value compounds. Diatoms are also able to produce large amounts of PUFAs, including eicosapentaenoic acid (EPA) and docosahexaenoic acid (DHA). Thus, the extraction of these PUFAs in combination with fucoxanthin can be an excellent approach that has to be further studied in marine microalgae [200,202].

#### 2.4.5. Current and Prospective Applications

Microalgal carotenoids have a wide range of applications, including in the cosmetic, pharmaceutical, food, and feed industries. Additionally, the development of aquaculture processes including microalgae as a nutritional supplement for fish can stimulate the production of microalgae in high-scale processes, with the reduction in cultivation and harvesting costs being one of the main objectives in microalgae biotechnology. Although the cost of producing synthetic carotenoids is lower than that of natural ones, the continuous increase in demand for natural products in the global market points to carotenoids produced by microalgae as highly demanded products in the following years. This, along with the sustainable production of microalgae, can make them outstanding candidates for the development of “green” biofactories in the near future [213].

#### 2.4.6. Research Needs

Although microalgae have a high potential for carotenoid production, further research is needed relating to the isolation, cultivation, extraction, or processing of algal biomass in order to reduce the cost of their industrial implementation. For example, isolating species from different extreme environments, such as high/low temperatures, high/low pH values, or high salinity, could be an interesting approach to finding strains able to produce higher amounts of carotenoids, such as lutein, fucoxanthin, or zeaxanthin. Additionally, genetic engineering is another strategy to improve the productivity of these compounds and achieve their economically feasible production. Reducing the cultivation costs, both by using alternative nutrient sources and improving the cultivation system, could also help boost productivity. This low productivity is one of the main problems in the industrial production of microalgae, which makes its competition with synthetic systems difficult. On the other hand, although there is much research about the use of emerging technologies (pressurized liquid extraction, pulsed electric fields, moderate electric fields, high-voltage electric discharges, high-pressure homogenization, microwave-assisted extraction, subcritical fluid extraction, and supercritical fluid extraction, among others) for the extraction of microalgal carotenoids [214,215], their industrial scaling-up remains a bottleneck. These concerns are the main challenge for future research in microalgal biotechnology to produce a more efficient and sustainable industry for the natural production of carotenoids from microalgae.

### 2.5. Yeast

#### 2.5.1. Ecological Importance

Yeasts are single-celled eukaryotic microorganisms that are members of the fungi kingdom. Since they are organotrophic and heterotrophic microorganisms, they require organic compounds as energy and carbon sources, and their growth is always associated with the presence of organic matter. Yeasts are globally distributed. They have been found in diverse habitats, including aquatic environments such as marine water, freshwater, glacier meltwater, groundwater, and the deep sea [216,217]. The concentration and diversity of yeasts in different aquatic habitats vary depending on the amount and type of organic matter and other environmental parameters. It has been observed that yeast populations decrease as the distance from the land increases. In coastal waters, thousands of yeast cells can be found per liter, while deep-sea regions can contain lower levels of yeast (10 cells/L, or even fewer) [20]. Despite the low numbers, the diversity is not low. For example, when studying the diversity of culturable yeasts in five locations in coastal waters on King George Island (maritime Antarctica), Garmendia et al. [218] identified eleven genera. The analysis by massive sequencing of the region ITS2 showed great diversity since 31 yeast genera were found. Kutty and Philip [20] reviewed studies on the diversity of marine yeasts around the world and found that the most frequently reported genera were *Candida*, *Cryptococcus*, *Debaryomyces*, and *Rhodotorula*. Of them, ascomycetous genera are predominant in shallow waters, and basidiomycetous yeasts predominate in the deep sea.

In both terrestrial and aquatic environments, yeasts play an important role as organic matter decomposers, facilitating the mineralization and recycling of nutrients. They also actively participate in nitrogen, sulfur, and phosphorous cycles [219]. Their ability to produce hydrolytic enzymes such as proteases, glycosidases, esterases, and lipases allows them to break down many organic polymers, producing lower molecular weight molecules that can serve as carbon or energy sources for themselves and for other organisms. In this way, their ecological role is very important in oligotrophic habitats. They also contribute to the bioremediation of contaminated areas due to their ability to decompose organic pollutants such as alkanes, phenolic compounds, or dyes [220,221]. Most yeasts undergo aerobic respiration, but some of them are also capable of obtaining energy by fermentation or anaerobic respiration using nitrate or nitrite as electron acceptors that produce NO or N_2_O, which are released into the atmosphere. In these cases, yeast would act as denitrifying microorganisms, causing the depletion of fixed nitrogen forms.

Some aquatic yeasts have evolved molecular and metabolic adaptations to survive or even grow under harsh conditions, such as low or high temperatures, high solar radiation, low nutrient availability, high salt concentrations, or even low or high pH [222]. In particular, in polar regions or at high altitudes, yeasts living in clear waters without natural shade are exposed to high UV radiation, which can influence diversity and population size [223]. UV radiation produces detrimental effects on living cells, causing direct or indirect damage to DNA, proteins, and lipids and accumulating ROS. To cope with such effects, some yeasts synthesize UV protectants and antioxidants such as carotenoids and accumulate them intracellularly [224]. The antioxidant properties of carotenoids may help extend the survival times of yeast in their natural habitats. Differences in growth between strains differing in carotenoid content showed a protective effect of those compounds [224].

#### 2.5.2. Sustainability Pros and Cons

Many aquatic yeasts can accumulate intracellularly high levels of carotenoids [225]; thus, they can be considered a natural and renewable source for carotenoid production.

As with other marine organisms, yeast, as a source of carotenoids, can be cultivated on agro-industrial waste or by-products in processes supporting the concept of a circular economy. As an example, the production of provitamin A carotenoids by *Rhodotorula glutinis* using goat cheese whey as a substrate has been recently described [226]. In addition, lycopene can also be obtained from yeasts by applying cheap agro-industrial waste such as flour extracts, whey, rice, and glycerol, among others [227]. In another interesting study, it was observed that the use of waste glycerol from the biodiesel production process in the yeast culture significantly increased the productivity and concentration of torularhodin compared to pure glycerol [228].

#### 2.5.3. Main Species (and Compounds) in the Context of Agro-Food and Health

A great diversity of carotenoids from different yeast species has been reported (Table 5). In most cases, various carotenoids can be obtained from a single yeast species. For example, as shown in the carotenoid database (http://carotenoiddb.jp, accessed on 30 May 2023), *Rhodotorula aurantiaca*, a psychrophilic red yeast isolated from Antarctica, can produce ten different carotenoids. Some yeast carotenoids can be found in other organisms. This is the case for β-carotene, which can be extracted from vegetables and fruits, or astaxanthin, which is also present in some microalgae, bacteria, crustaceans, or fish such as salmon and trout [229]. However, other yeast carotenoids, such as torulene and torularhodin, are specific fungal metabolites.

Yeasts that accumulate remarkable levels of carotenoids are basidiomycetous yeasts belonging to the Cystofilobasidiales and Sporidiobolales orders. Among Cystofilobasidiales, only one species of *Phaffia rodozyme* (teleomorph *X. dendrorhous*) has been reported to be carotenogenic [230]. This species, which has been isolated from some tree exudates, leaves, fungal stromata, and freshwater [231], accumulates astaxanthin, a carotenoid with many biotechnological applications [230]. Among Sporidiobolales, yeasts that accumulate the highest levels of carotenoids belong to the genera *Rhodotorula* and *Sporobolomyces* and to their teleomorphs, *Rhodosporidium* and *Sporidiobolus* [37]. The carotenoid profile produced by these yeasts is mainly represented by γ-carotene, β-carotene, torulene, and torularhodin [232], but other compounds have also been described. Carotenogenic yeasts from Sporidiobolales have been isolated from diverse aquatic habitats. Ueno et al. [225] isolated 40 yeast strains from different aquatic environments in Japan. These yeasts accumulated carotenoids in amounts greater than 200 µg/g of their dry cell mass. They were identified as *Rhodotorula* spp. and *Rhodosporidium* spp. and produced different amounts of intracellular β-carotene, torularhodin, γ-carotene, torulene, and β-zeacarotene, the first three compounds being the most abundant. Carotenogenic isolates from different species within the *Rhodotorula* genus have been isolated from the coastal waters of King George Island (maritime Antarctica) [218] and from oligotrophic lakes in Patagonia, Argentina [233]. Pigmented *Rhodotorula* spp. strains that accumulated intracellular carotenoids were also isolated from seawater near Chile [234]. One of the strains accumulated carotenoids, representing 19% of its dry weight, when cultured in glycerol as the only carbon source. The carotenoid profile was not very common and included β-carotene, adonirrubin, 3-hydroxyechinenone, and canthaxanthin. Strains from Sporidiobolales identified as *R. mucilaginosa*, *R. toruloides*, *R. diobovata*, and *S. roseus* recognized as species that accumulate significant amounts of carotenoids [235] were also found in oligotrophic hypersaline coastal waters of the Arabian Gulf [236].

Carotenoids are also produced in fewer amounts by basidiomycetous yeasts of the Sporidiobolales order. Strains belonging to different species from the genera *Bullera*, *Cystobasidium*, *Cystofilobasidium*, and *Dioszegia*, many of which have been isolated from aquatic environments, produce intracellular carotenoids [237]. Most of the compounds found in these yeasts are carotenes. However, two uncommon xanthophylls, 16-hydroxytorulene and torularhodinaldehyde, which were previously known only from chemical synthesis, have been found in certain *Cystofilobasidium* species [238]. Moreover, some isolates belonging to the genus *Dioszegia* produce almost exclusively plectaniaxanthin, a xanthophyll that is a dihydroxylated derivative of torulene [239]. These carotenoids represent a minor fraction of the carotenoids produced by yeast species but are illustrative of their chemical diversity.

Undoubtedly, yeasts from aquatic environments throughout the world are a source of common and rare carotenoids that should be further explored.

Many biological properties have been demonstrated for the main yeast carotenoids, which include carotenes such as β-carotene, γ-carotene, and torulene, and xanthophylls, including astaxanthin, canthaxanthin, and torularhodin [240]. Some of them, such as γ-carotene, β-carotene, torulene, and torularhodin, are vitamin A precursors [241]. Anti-bacterial and anti-fungal activity has been demonstrated for torularhodin, a specific fungal carotenoid [242,243]. Moreover, due to its important antimicrobial activity, it was successfully used to protect implanted medical devices against microbial contamination [244,245]. In the case of torulene and torularhodin, Du et al. [246] postulated that their action in the inhibition of prostate cancer in nude mice was associated with the apoptosis of tumor cells.

**Table 5:** Examples of carotenoid content in marine yeast.

Species	Carotenoids	Collection Sites	Experimental Conditions	Quantification/Identification Methodologies	Contents	References
*Sporidiobolus salmonicolor*	β-carotene, 2,3 dihydroxy-γ-carotene, 4-ketotorulene, and torulene	Union Glacier, Antarctic	30 mL YM broth in 300-mL baffled flasks, 150 rpm, 20 °C, 5 days	HPLC	2,3-dihydroxy-γ-carotene was the main carotenoid	[247]
*Sporidiobolus metaroseus*	β-carotene, β-cryptoxanthin, 4-ketotorulene, and spirilloxanthin	Union Glacier, Antarctic	30 mL YM broth in 300-mL baffled flasks, 150 rpm, 20 °C, 5 days	HPLC	β-carotene and 4-ketotorulene were the main carotenoids	[247]
*Rhodotorula mucilaginosa*	Astaxanthin, β-carotene, and lycopene	Chiloe, 30 km southeast of Puerto Montt, Chile	4 g yeast/L seaweed (25% *v*/*v*), 150 rpm, 25 °C, 6 days	HPLC	1.84 ± 0.03 mg/L total carotenoids. Carotenoids proportion: 1.8 ± 0.3% astaxanthin, 21.8 ± 1.5% β-carotene, and 38.4 ± 9.4% lycopene	[248]
*Rhodosporidium babjevae*	β-carotene, γ-carotene, torularhodin, and torulene	Grøtsundet, Northern Norway	10 g/L Difco Marine broth 2216 (10 g/LDifco Bacto-peptone, 10 g/L glucose, 15 g/L NaCl, and 15 g/L agar), 6 °C, 140 h	HPLC	Torularhodin > torulene > β-carotene > γ-carotene	[249]
*Rhodotorula mucilaginosa*	β-carotene, torularhodin, and torulene	Escondido lake, North-western Patagonia, Argentina	20 mL of minimal medium salt broth (MMS), 25 °C, 250 rpm, 24 h	TLC (for pigment profile), spectrophotometry, and HPLC	205 ± 15 µg/g total carotenoids, DW. Carotenoid proportions: 10.8% β-carotene, 83.4% torularhodin, and 5.7% torulene	[250]
*Rhodotorula dairenensis*	β-carotene, γ-carotene, torularhodin, and torulene	Freshwater in the middle of the Sagamigawa River, Japan	20 mL of YPD liquid medium (2% glucose, 2% peptone, and 1% yeast extract), 25 °C, 120 rpm, 36 h	HPLC	267 µg/g total carotenoids, DW	[225]

DW, dry weight; HPLC, high-performance liquid chromatography; TLC, thin-layer chromatography.

#### 2.5.4. Production Approaches and Production Data in Different Continents

The production of carotenoids by yeast cultures can be considered a sustainable process. Although the use of yeast for carotenoid production is not economically profitable yet, efforts to improve yields and reduce production costs have merged to meet global demands for the use of natural products.

The biosynthesis of carotenoids by yeast is influenced by many factors that can modify yields and affect operating costs. The yeast strains, culture medium composition (type and concentration of nutrient sources), and growth conditions (temperature, pH, oxygen, and light) influence the amount and profile of carotenoids accumulated within the yeast cell [251,252] (Table 5).

Carotenoid biosynthesis in yeast begins in the late logarithmic phase and continues in the stationary phase; thus, the optimal harvest time to obtain the maximum content of carotenoids should be determined precisely in each case. The type and concentration of the main nutrients, such as carbon and nitrogen sources, are also important factors that significantly regulate the carotenogenesis process. Many studies on carotenoid synthesis in yeast cultures have been conducted in synthetic media with pure carbon sources, such as glucose, sucrose, xylose, cellobiose, glycerol, or a combination of some of them [253]. To lower production costs and in the search for circular economy strategies, many by-products, wastes, and raw materials from agro-industries, such as raw glycerol, brewery effluents, molasses, grape must, and milk whey, have been proposed as alternative carbon sources for carotenoid production [226,254,255,256].

An important fact for obtaining high levels of intracellular carotenoids is to consider that carbon sources cannot be the limiting factor in yeast growth. However, high initial concentrations of sugars are detrimental to carotenoid accumulation in batch cultures of yeasts that exhibit the Crabtree effect, such as *Phaffia rodozyma* [257]. In these cases, at high concentrations of carbon sources, fermentation occurs even under aerobic conditions. In consequence, pyruvate from glycolysis is partially transformed in ethanol and not totally derived to acetyl-CoA, which is a precursor for the mevalonate pathway, the first step in the carotenoid biosynthesis pathway [256]. The best strategy to improve biomass and carotenoid production under such conditions is the implementation of fed-batch cultures [258].

The C/N ratio is an important parameter to optimize carotenoid accumulation in yeast. Much work related to this topic has been published; however, there are no general rules to follow. Although it is fairly agreed that high C/N ratios improve carotenoid synthesis [257], there are some cases in which the results are different [259]. Differences in carotenoid profiles have also been observed with different C/N ratios [29].

In the case of *R. glutinis*, the effect of the C/S and C/P ratios on the accumulation of carotenoids has also been studied [252,260]. In both cases, an increase in the sulfur and phosphorus source concentrations improved the production of carotenoids.

Light also influences the production of carotenoids by yeast. To avoid possible damage caused by light exposure, carotenogenesis is induced when yeasts grow in the presence of light. The effect of light depends on the yeast strain and is related to an increase in the activity of certain enzymes associated with the carotenoid biosynthetic pathway [261]. Light induction of carotenoid biosynthesis has been reported for different yeast species such as *R. glutinis* [262], *Rhodosporidium toruloides* [263], and *P. rodozyma* [264].

Another factor that influences the production of carotenoids by yeasts is temperature. Studies on the effect of temperature on carotenoid content in *Rhodosporidiobolus colostri* [265], *Rhodotorula babjevae* [249], *R. mucilaginosa* [266], and *R. glutinis* [267] coincide in that at higher growth temperatures an increase in torularhodin is obtained in detriment of the β-carotene content. The effect on torulene varied with the specific yeast strain. According to Zhao and Li [267], the enhancement in torularhodin production at higher temperatures might be due to its high antioxidant activity and the protection it would exert by scavenging ROS induced by high-temperature stress.

Carotenogenic yeasts are mainly aerobic microorganisms. The oxygen supply is an essential factor to enhance their growth in culture conditions, and it influences the amount and profile of carotenoids accumulated inside the cells [268]. Oxygen levels influence the relative concentration of carotenes and xanthophylls since the latter are formed through the oxidation of the former [29]. The synthesis of astaxanthin by *P. rodozyma* is greatly stimulated by oxygen supply. In the presence of low oxygen levels, yellowish biomass is formed due to the accumulation of β-carotene [258].

The yeast *P. rodozyma* accumulates intracellular astaxanthin but in much lower amounts than the microalgae *Haematococcus pluvialis*, so its commercial production is not as extensive. At present, there is at least a commercial product called Astaferm based on astaxanthin obtained from *P. rodozyma*. It is produced for human consumption in various presentations by the company Nextferm (Israel) and is also sold in the USA. Information about these products can be found on the company website (https://www.nextferm.com/, accessed on 30 May 2023).

#### 2.5.5. Current and Prospective Applications

Currently, the carotenoids found in yeasts have many commercial applications. However, their production at the industrial level, in most cases, does not involve their extraction from yeasts. The main reasons for that are the low yields of the processes and the high costs associated with microbial production and product purification. Astaxanthin, a xanthophyll produced by *P. rodozyma*, is a clear example of it. Astaxanthin is a carotenoid extensively used in aquaculture as a feed additive for imparting reddish coloration to the flesh of salmon, trout, shrimp, and ornamental fish [229]. It is also used in poultry to enhance the color of egg yolks and the skin and meat tissue of broilers, resulting in better acceptance in the market [269]. Due to its various health benefits, astaxanthin has been incorporated into food and beverages, nutraceutical preparations, and cosmetics [229]. Its industrial production is mainly based on chemical synthesis, except for products devoted to human direct consumption, for which natural astaxanthin is preferred [36].

Other yeast carotenoids, such as torulene and torularhodin, are not currently produced or used in industry; however, they have many potential applications. They have a reddish color and could be used as pigments in animal feeding in aquaculture or poultry [270]. Due to their proven activities as antioxidants and vitamin A precursors, they could also have applications as food and cosmetic additives. Moreover, both carotenoids have been associated with tumor prevention, so they could also be postulated as nutraceutical products [240]. These carotenoids are synthesized only by yeast and fungi, and their effect on humans and animals is still unknown.

#### 2.5.6. Research Needs

The carotenoids present in yeasts have the potential to be used as food and cosmetic ingredients, as well as pigments in aquaculture and poultry. However, much research is still needed so that the production of carotenoids in yeast is competitive in the market. Some yeast carotenoids, such as torulene and torularhodin, have not been used in animals yet due to fewer studies on their safety and effects on health [240]. Further research is also needed to determine the properties of other carotenoids produced by aquatic yeasts that have not been explored yet. On the other hand, the extraction of yeast carotenoids has been scarcely studied.

## 3. Roles of Carotenoids in Marine Organisms

Carotenoids are biosynthesized by all photosynthetic organisms (plants, algae, and cyanobacteria), as well as some fungi, non-photosynthetic archaea, bacteria, and arthropods. The vast majority of animals obtain them through their diet [35].

### 3.1. Photosynthesis

Carotenoids are essential in photosynthesis, intervening in actions such as light harvesting, energy transfer, photoprotection, or the assembly of pigment-protein complexes in the photosynthetic apparatus. Some microalgae can biosynthesize large amounts of carotenoids in response to stress conditions. They are deposited in lipid globules that can be found outside the photosynthetic apparatus (for instance, astaxanthin produced by *Haematococcus*) or within it (as appears to be the case for the massive β-carotene accumulation observed in some *Dunaliella* species). Carotenoids are also thought to somehow intervene in photoreception and phototaxis in some flagellate microalgae, which have a photosensitive eyespot capable of sensing variations in light intensity. The photoreceptors are usually membrane-bound rhodopsins (consisting of an apoprotein bound to retinal, a carotenoid derivative) located close to layers of photoprotective/reflective carotenoid-rich globules. Photoreception and phototaxis are important in deciding where to go for optimal light or to prevent predation [271].

There is a diverse body of evidence that carotenoids can protect aquatic organisms from various stressors. As can be inferred from the information provided below, some of the possible mechanisms could be protection against oxidation and modulation of membrane properties.

### 3.2. Secondary Antenna in Retinal Proteins

The important role that carotenoids can play as secondary antennas of type I rhodopsins has recently been demonstrated [272]. Many marine and freshwater bacteria, as well as some eukaryotes, are capable of obtaining energy using sunlight, thanks to type I rhodopsins. These proteins, evolutionary-divergent but structurally related to the rhodopsin II of animals, have retinal as their main chromophore. The retinal of the rhodopsin proteins and the chlorophylls of the photosystems are the only two types of pigments with the ability to convert solar energy into chemical energy that, to date, are known. Since the discovery of the first type I rhodopsin in the 1970s, bacterioruberin, which pumps H^+^ thanks to the sunlight, generating a proton-motive force used by ATP synthase for the production of ATP, many other similar light-driven pumps have been discovered [273,274].

In 2005, it was announced for the first time that a carotenoid, the keto-carotenoid salinixanthin, could act as a secondary antenna for a retinal rhodopsin. The study showed that salinixanthin, found in the halophile bacteria *Salinibacter ruber*, is able to harvest light energy and transfer it to retinal [275]. The recent discovery that the hydroxylated carotenoids, lutein and zeaxanthin, can also act as secondary antennas demonstrates that the ability of salinixanthin is not an exceptional case and uncovers a new essential role of carotenoids as auxiliary antennas in retinal rhodopsins. Furthermore, the study estimates, on the basis of the sequence and structural features of many known rhodopsins, that carotenoids could be the second chromophore in about half the microbial retinal rhodopsin proteins, having an essential role in the microbial photoautotroph metabolism in the oceans and continental waters [272].

### 3.3. Oxidative Stress

*Halobacillus halophilus* is a moderately halophilic bacterium that can tolerate up to 3 M sodium chloride. It accumulates C30 carotenoids structurally related to staphyloxanthin that impart a yellow hue. By using diphenylamine (an inhibitor of the biosynthesis of colored carotenoids) and the oxidant duroquinone, it was shown that the C30-colored carotenoids were essential for the survival of the bacteria under oxidative stress conditions [276].

Cyanobacteria that fix nitrogen (diazotrophs) are especially susceptible to reactive oxygen species due to the sensitivity of nitrogenase to oxygen. The total antioxidative potential of various diazotrophic cyanobacteria (*Cyanothece*, *Anabaena*, and especially *Trichodesmium* spp., a bloom-forming cyanobacteria that contributes to a great extent to global nitrogen fixation) and non-diazotrophic cyanobacteria (*Prochlorothrix*, *Synechococcus*) has been evaluated by means of the ferric reducing/antioxidant power (FRAP) assay. To gain insight into the different cell components that contribute to total antioxidant activity, non-polar and polar organic extracts as well as crude protein extracts were tested. *Trichodesmium* sp. IMS101 showed the highest activity among the samples tested. The relative contribution to the total antioxidant activity ranged from 54% for the non-polar organic extract to only 13% for the protein extract, a result that contrasted with the average relative contribution of each extract of the other cyanobacterial species tested (86% for the protein extract, 9% for the non-polar organic extract, and 5% for the polar organic extract). As a result of subsequent studies (bioassay-guided fractionation and HPLC profiling of purified fractions), the authors pinpointed β-carotene and retinyl palmitate as possible molecules contributing to the high in vitro antioxidant activity of the non-polar organic extract obtained from *Trichodesmium* sp. IMS101 [277].

### 3.4. Salt Stress

Halophilic archaea or haloarchaea are extremophiles that require high salt concentrations for optimal growth. They can be found in saline environments, including salt lakes, solar salterns, and salted foods. The predominant carotenoids present in them are C50 carotenoids, such as bacterioruberin and its derivatives, monoanhydrobacterioruberin and bisanhydrobacterioruberin [278].

Carotenoid-rich microbial communities are well known to occur in solar salterns where there are environments with different salt concentrations, ranging from seawater to NaCl saturation. In crystallizer ponds, the green microalga *Dunaliella* appears as the sole primary producer. It occurs together with dense communities of heterotrophic halophilic archaea that thrive due to the carbon photosynthetically fixed by the microalga. The red hue of the crystallizer ponds increases light absorption, raises the temperature, and enhances salt production. The coloration is due to the massive accumulation of β-carotene by *Dunaliella* and the carotenoid and retinal protein-based pigments of the Archaea (for example, *Haloquadratum walsbyi* and other *Halobacteriaceae* or *Salinibacter*) [279].

Bacterioruberin and other archaeal C50 carotenoids are believed to increase membrane rigidity and provide protection against UV light. The presence of carotenoids in the membrane could help microbes cope with hypersaline conditions by acting as a water barrier and allowing the passage of ions and oxygen molecules through the cell membrane. Bacterioruberin has also been shown to be a component of specific transmembrane proteins and modulate membrane dynamics and physics [19].

### 3.5. Low Temperatures

The cryosphere has been colonized by a variety of microbes collectively known as psychrophiles. Temperatures close to 0 °C lead to increased viscosity of solvents and solubility of gases, reduced solubility of nutrients and solutes, decreased diffusion and amplified desiccation, osmotic stress, and ice development. Other characteristics of these environments are high salinity, low water activity and availability of nutrients, oxidative stress, freeze–thaw cycles, extremes of light (high at high altitudes and low in frosted lakes, permafrost, and deeper ice sheets), and exposure to UV radiation. Among the strategies that cold-adapted microbes have developed to cope with these harsh conditions is the production of pigments, which are very often carotenoids [280].

The biosynthesis of carotenoids (haloxanthin, monoanhydrobacterioruberin, bacterioruberin, bacterioruberin monoglycoside, and bacterioruberin diglycoside) of the Antarctic psychrotrophic bacterium *Micrococcus roseus* was shown to increase at 5 °C compared to 25 °C; additionally, more polar carotenoids (the two glycosides) were produced at 5 °C [281].

The carotenoid content of the Antarctic psychrotolerant bacterium *Sphingobacterium antarcticus* and the mesophilic bacterium *Sphingobacterium multivorum* has been studied, revealing that the main carotenoids were zeaxanthin, β-cryptoxanthin, and β-carotene. Interestingly, the relative amounts of polar pigments were higher in cells grown at 5 °C than in cells grown at 25 °C, and the synthesis of polar carotenoids was higher in the psychrotolerant strain [282].

*Arthrobacter* is a ubiquitous bacterial genus. Some *Arthrobacter* species produce the rare C50 carotenoid bacterioruberin, which is found mainly in haloarchaea. Interestingly, strains of *A. agilis* and *A. bussei* exhibited increased bacterioruberin biosynthesis when the culture temperature was reduced from 30 to 10 °C. The increased levels of bacterioruberin were correlated with increased membrane fluidity at low-temperature growth and with improved cell resistance to freeze–thaw stress [283]. In another study, heterotrophic Antarctic bacteria were subjected to freeze–thaw cycles and simulated solar radiation, and a higher survivability was observed in carotenoid-pigmented bacteria [77].

### 3.6. High Temperatures

Bacteria of the genus *Thermus* can also tolerate high temperatures. *Thermus thermophilus* is known to accumulate uncommon carotenoid (specifically zeaxanthin) diglucoside esters and monoglucoside esters, generically termed thermozeaxanthins. In thermophilic bacteria, carotenoids span the membrane and are believed to stabilize it by modulating its fluidity, which is essential for its survival in such harsh conditions. This hypothesis is supported by experiments in which thermozeaxanthins were incorporated into egg phosphatidylcholine liposomes, resulting in their stabilization in the temperature range of 30–80 °C. On the other hand, polar carotenoids in artificial membranes can reduce the rate of oxygen diffusion and protect membrane lipids from oxidation [284].

*Blastochloris tepida* is a recently described thermophilic purple bacterium. In an interesting experiment, the light-harvesting one reaction center core complexes of this species were compared with those of its mesophilic counterpart, *B. viridis*. Both a higher total carotenoid content and a different carotenoid profile (with higher levels of carotenoids with more than nine conjugated double bonds) were detected in *B. tepida.* Additionally, it was observed that thermostability decreased when *B. tepida* was treated with diphenylalanine, an inhibitor of the biosynthesis of colored carotenoids. Altogether, the results indicated that *B. tepida* carotenoids are important for high-temperature tolerance [285].

### 3.7. Roles in Marine Animals

The carotenoids of marine algae and microbes are incorporated into herbivorous marine animals (e.g., sponges, sea anemones, bivalves, microcrustaceans, and tunicates) and can be modified through metabolism; then these herbivores serve as foods for carnivorous animals, such as snails, crustaceans, starfish, and fish. Thus, in marine animals, it is common to find metabolites of β-carotene, fucoxanthin, peridinin, diatoxanthin, alloxanthin, and astaxanthin [286]. Concerning the role of carotenoids in marine animals, those with an unsubstituted β-ring (for instance α-carotene, β-carotene, or β-cryptoxanthin) can be converted into vitamin A. Unlike in humans, carotenoids with substituted β-rings (canthaxanthin, lutein, zeaxanthin, and astaxanthin) have been reported to be precursors of vitamin A forms in some fish [286]. In addition to their presumed antioxidant and immunomodulatory activities, carotenoids are usually found in integuments, where they can be important for photoprotection, camouflage, and signaling for breeding. Besides, carotenoids are common in marine animal gonads, and there is evidence that they are important for reproduction (for instance, in ovary development, fertilization, hatching, or larval growth and survival) [286].

## 4. Carotenoids: Versatile Compounds with Health-Promoting for Foods, Cosmetics and Other Products

The importance of carotenoids for food security is beyond any doubt, as they are essential for photosynthesis, the engine of life on Earth, and the primary driver of food production. Besides carotenoids and/or apocarotenoids (i.e., compounds obtained by the carotenoid cleavage, such as many volatiles, the phytohormones strigolactones, and abscisic acid), they are important for pollination, seed dispersal, plant resilience to diverse stresses, etc. Their roles as colorants and, for some of them, as vitamin A precursors have been long known. Since the last three decades, there has been an expanding interest in their possible health-promoting biological actions, which could also be attributed, at least to some extent, to apocarotenoids [33,35]. The possible mechanisms are several: direct antioxidant actions (quenching, scavenging), pro-oxidant actions, enhancement of gap junctional intercellular communication, modulation of signaling pathways or immune function, and absorption of visible light (or UV in the case of the colorless carotenoids phytoene and phytofluene), which may interact. As a result, different effects such as antioxidative, prooxidative, anticarcinogenic, or anti-inflammatory can take place. Thus, carotenoids are thought to contribute to reducing the risk of cancer, cardiovascular diseases, metabolic, bone, skin, or eye disorders. Positive effects on cognitive performance and during pregnancy and early life are also being increasingly reported [33,35,287,288,289]. They can also provide aesthetic benefits as, together with melanin and hemoglobin derivatives, they are contributors to skin color and may have other cosmetic benefits. Therefore, carotenoids can be used in products for human consumption (foods, supplements, nutraceuticals, nutricosmetics, etc.) as colorants, vitamin A precursors, antioxidants, and health-promoting compounds [33,34,290]. Research on carotenoids has been propelled in recent years by cohesive and functional research networks, such as IBERCAROT in Ibero-America (http://www.cyted.org/?q=es/detalle_proyecto&un=829, accessed on 30 May 2023), EUROCAROTEN in Europe (https://www.cost.eu/actions/CA15136/#tabs|Name:overview, accessed on 30 May 2023) or the Spanish Carotenoid Network (CaRed).

## 5. Potential Health-Promoting Actions of Carotenoids from Aquatic Organisms

In human physiology, ROS are used as important signaling molecules, but their production has to be tightly controlled. Unbalanced ROS generation, resulting in oxidative stress and closely related to inflammatory signaling, contributes to many chronic diseases [291,292]. Although, as has already been commented, carotenoids, including those from marine organisms, can be involved in different actions contributing to health promotion, much attention is paid to their possible antioxidant and anti-inflammatory effects. Astaxanthin, torulene, and torularhodin, which are carotenoids commonly found in marine organisms, have been shown to exhibit higher antioxidant capacity than β-carotene under certain conditions [29,229]. In addition, anticancer activity has been demonstrated for these carotenoids [246,293,294,295]. Among the carotenoids of marine organisms, there are some, such as astaxanthin, that have been shown to exhibit both antioxidant and anti-inflammatory activity, and thus they are considered potential health-promoting agents against atherosclerotic cardiovascular disease [296]. Astaxanthin also exhibits antimicrobial activity. Its antifungal and antibiofilm activity against *Candida albicans* and *Candida glabrata* has recently been reported [297]. Studies in this respect involving “marine carotenoids” are summarized in Table 6. Only a few examples are included in this table, but extensive works can be found in the literature on some marine microorganisms, such as microalgae and seaweed [298,299].

## 6. Advantages and Disadvantages of Using Marine Organisms as a Source of Carotenoids over Chemical Synthesis

In general, microorganisms, including those of marine origin, have unique benefits for carotenoid production, such as their short life cycle, adaptability to various seasons and climates, the ability to generate a diverse range of carotenoids with varying colors and biological properties, and easier scalability of production [320]. Some of these characteristics can be attributed to other maritime organisms, such as seaweeds. In fact, the microalgae *Dunaliella salina* and *Haematococcus pluvialis* have been long used for the commercial production of β-carotene and astaxanthin, respectively. Besides, yeasts of the Rhodotorula genus and bacteria of the *Flavobacterium* genus, just to mention a couple of examples, elicit increased interest in the commercial production of carotenoids, highlighting the current and future competitiveness of fermentative processes for the carotenoid market [321].

One of the main drawbacks of using carotenoids from natural sources directly from the matrices (without prior isolation/extraction) is their frequently lower bioavailability relative to formulated products. This is because the matrix must be broken down during digestion before the carotenoid component can be released and utilized. This common low bioavailability of carotenoids present in natural sources can result in low bioactivity. For example, in a study by Edgar et al. [322] in which the resistance of rainbow trouts to a viral pathogen after oral ingestion of synthetic carotenoids (β-carotene, astaxanthin, and canthaxanthin) or natural sources of carotenoids (such as *Dunaliella salina* and *Phaffia rhodozyma*) was examined, it was observed that mortality was markedly reduced in the fish fed with astaxanthin, while it was only slightly reduced (with no statistical difference) in those fed with *D. salina*. Nonetheless, in the last few years, modern processing techniques have been used to overcome these limitations, leading to a rise in commercially available natural sources [322].

On the other hand, an advantage of using the complete matrix as a source of carotenoids is that it can contain other bioactive compounds. These compounds may also be present in carotenoid extracts obtained from natural sources, which is not the case with synthetic carotenoids. As an example, β-carotene from *Dunaliella* contains numerous carotenoids and essential nutrients that are not present in synthetic β-carotene [323].

In the production of carotenoids, it is important to consider the isomers of the carotenoids that are obtained, since different isomers of a carotenoid may have different bioavailability and biological activity. In this regard, it should be noted that, in some cases, chemical synthesis would have to be optimized in order to be able to isolate only those isomers with greater bioavailability or biological activity, which could be obtained from natural sources. This optimization could increase the cost of production. Astaxanthin represents a clear example of the advantages of obtaining a carotenoid from natural sources. Natural astaxanthin, for example, from *H. pluvialis* and *Paracoccus*, occurs in the trans form (3*S*, 3*S*), whereas the synthetic production of astaxanthin generally obtains an isomeric mixture of (3*S*,3′*S*), (3*R*,3′*S*), and (3*R*,3′*R*) in a ratio of 1:2:1. The separation of the active isomer from the synthetic mixture could increase production costs. In several studies, the in vitro or biological activity of the isomeric mixture of astaxanthin synthetically produced has been shown to be lower than that of the carotenoid obtained from natural sources. Lastly, natural astaxanthin has been reported to have higher stability and better bioavailability compared to some of the stereoisomers obtained synthetically [36,324]. In relation to fucoxanthin, some authors indicate that, although this carotenoid can be synthesized chemically, its extraction from brown seaweed is a more accessible, safe, and economical method. However, the content in brown seaweed varies greatly by species, geographical location, season, temperature, salinity, light intensity, and interactions among these factors [325].

Another aspect to consider is the presence of carotenoid esters naturally present in some marine organisms. Generally, carotenoid esters have a higher stability than the respective free carotenoids. As an example, in microalgal extracts were found ca. 70% astaxanthin-monoesters, 25% astaxanthin-diesters and 5% free astaxanthin [326]. In relation to chemical synthesis, the esterification of carotenoids could lead to an increase in the price of production.

It is also important to consider that both synthetic and natural carotenoids must pass strict analyses before they are allowed for human consumption. In this regard, both chemical synthesis and isolation from natural sources could generate undesirable compounds that should be eliminated from the final product. In some cases, for human consumption, it has been preferred to opt for natural sources instead of chemical synthesis. This is the case with astaxanthin. The natural form of this carotenoid was introduced as a human nutraceutical supplement in the late 1990s, following approval by the Food and Drug Administration (FDA) as a new dietary ingredient, while synthetic astaxanthin has not been officially registered for direct human use in any country by regulatory authorities and has been used predominantly for animal feed [209,320].

Another advantage of using marine organisms as sources of carotenoids is related to consumer acceptance. Consumers generally feel that synthetic compounds are less safe than those obtained from natural sources. For example, in a recent study, it was observed how chemicals of natural origin are considered to be healthier and safer than synthetic ones and how consumers frequently associated the word “synthetic” with “unnatural”, “health hazard”, and “environmental hazard” [327]. As a consequence, some consumers are willing to pay high prices for products of natural origin [320]. However, as mentioned earlier, one approach to enhancing the productivity of marine organisms as a carotenoid source is through genetic engineering. In this regard, it is important to note that a considerable portion of consumers who oppose the consumption of synthetically derived carotenoids are likely to hold similar reservations about consuming carotenoids obtained from genetically modified organisms.

The increased demand for natural carotenoids as food additives has spurred advancements in technological innovations for the manufacturing of microbial carotenoids. Numerous research studies have emphasized sustainable and biocompatible methods for manufacturing carotenoids from natural sources, promoting the use of safer carotenoids obtained through alternative means rather than chemical synthesis. The retro-sustainable biotechnological pathway can be accomplished through the implementation of a circular economy approach. This approach not only enables carotenoid recovery but also the possibility of obtaining additional value-added substances such as lipids and proteins associated with the recycling of raw materials for bioproduction and solvents used in extraction procedures. On the other hand, it is frequently thought that the production of synthetic carotenoids does not follow the principles of the circular economy and is destructive to the ecosystem. Thus, synthesis using nonrenewable sources reveals a negative impact on the environment and consumers [320].

However, there are important hurdles to overcome in order to increase the use of marine organisms to extract carotenoids instead of obtaining carotenoids from chemical synthesis. The main barriers are the acceptability by some consumers of new sources, the low bioavailability of carotenoids from natural sources (which can result in low bioactivity), and, of course, the safety of the product as food ingredients. Several research studies are needed, in particular about the metabolization of carotenoids from natural sources by humans and their health benefits, although there are many others. Further, the extraction of carotenoids from natural origin cannot yet compete in price with the chemical synthesis of carotenoids and could still present some negative environmental impacts. Thus, strategies and technologies (e.g., genetic engineering, CRISPR, fine-tuned cultivation conditions, sustainable and scalable extraction methodologies, and green solvents for biorefineries) may therefore be required to increase the production and use of carotenoid-rich natural products.

## 7. Concluding Remarks

Altogether, the organisms investigated in this review can accumulate both common dietary carotenoids (β-carotene, lutein, zeaxanthin, astaxanthin, canthaxanthin, or fucoxanthin in some countries) and unusual ones (e.g., bacterioruberin, salinixanthin, myxoxanthophyll, and β-zeacarotene), some of which are unique to some taxa. The fact that some studies provide evidence that unusual “marine carotenoids” exhibit important properties such as distinctive colors, high in vitro antioxidant capacity, or even health-promoting biological actions in studies of diverse nature is encouraging.

Some advantages of using marine organisms as a source of carotenoids over chemical synthesis are that, in some cases, chemical synthesis produces isomers with lower activity, bioavailability, and stability. In addition, marine organisms have a great capacity to synthesize a wide variety of carotenoids, which can be obtained in a renewable manner without depleting natural resources. Thus, marine organisms have the potential to be key sustainable sources of these compounds, which could help Europe achieve its Green Deal and Recovery Plan. However, to consolidate the industrial production of carotenoids from marine organisms, more research is needed to increase productivity and reduce costs. In summary, the need to promote the blue economy to help produce sustainable and health-promoting foods offers an exciting opportunity to tap into aquatic ecosystems and valorize carotenoid-rich organisms.

## Figures and Tables

**Table 1:** Examples of carotenoid content in marine archaea.

Species	Carotenoids	Collection Sites	Experimental Conditions	Quantification/Identification Methodologies	Contents	References
*Halobacterium halobium*	Total carotenoids (mainly bacterioruberin)	Saltern brine, Sfax, Tunisia	Liquid culture (10 g/L yeast extract, 7.5 g/L casamino acids, 250 g/L NaCl, 20 g/L MgSO_4_.7H_2_O, 2 g/L KCl, and 3 g/L trisodium citrate), 37 °C, 240 rpm, 7 days	Spectrophotometry	5.66–7.63 mg/L, FW	[48]
*Haloarcula* *japonica*	Total carotenoids (mainly bacterioruberin)	Saltern soil	Liquid culture, 37 °C, 10 days	HPLC-MS	335 μg/g, DW. Bacterioruberin was up to 68.1% of the total carotenoids (mol%)	[49]
*Halorubrum* sp.	Total carotenoids (mainly bacterioruberin)	Saltern brine	Liquid culture, 37 °C	Spectrophotometry	25 mg/L, FW	[50]
*Haloterrigena turkmenica*	Total carotenoids (mainly bacterioruberin)	Salt soil crust	Liquid culture, 37 °C, 180 rpm	Spectrophotometry	74.5 μg/g, DW	[51]
*Halorubrum* sp.	Total carotenoids (mainly bacterioruberin)	Seven distinct saline habitats, Algeria	Liquid culture, 37 °C	Spectrophotometry	3.68 mg/L, FW	[52]
*Haloarcula* sp.	Total carotenoids (mainly bacterioruberin)	Tebenquiche Lake of the Atacama Saltern, Chile	Liquid culture (5 g/L proteose-peptone, 10 g/L yeast extract, 1 g/L glucose, with 25% (*w*/*v*) total salts), 40 °C, 120 rpm, 10 days	HPLC-MS	488.88–871.53 mg/g, DW	[53]
*Haloferax mediterranei* strain R-4 (ATCC33500)	Total carotenoids (mainly bacterioruberin)	Salt pond	Liquid culture, 36.5 °C	Spectrophotometry	92.2 µg/mL, FW	[54]

DW, dry weight; FW, fresh weight; HPLC, high-performance liquid chromatography; MS, mass spectrometry.

**Table 6:** Some examples of carotenoids in marine organisms with potential antioxidant and anti-inflammatory health-promoting activities.

Organisms	Species	Carotenoids	Biological Activities	Methodologies	References
Archaea	*Halobacterium halobium*	Bacterioruberin ^1^	Antioxidant	Exposing hepatoma cell lines with carotenoid extract to arachidonic acid or H_2_O_2_. The protective effect against oxidative stress was also assessed by the MTT assay	[48]
	*Haloarcula japonica*	Bacterioruberin ^1^	Antioxidant	DPPH assay	[49]
	*Haloterrigena turkmenica*	Bacterioruberin ^1^	Antioxidant	DPPH and FRP assays	[51]
	*Haloferax volcanii*, *Halogranum rubrum*, *Haloplanus inordinatus*, *Halogeometricum limi*, *and Haloplanus vescus*	Bacterioruberin ^1^	Antioxidant	DPPH assay and the evaluation of the inhibition of H_2_O_2_-induced hemolysis of mouse erythrocytes	[57]
	*Haloarcula hispanica* and *Halobacterium salinarum*	Bacterioruberin ^1^	Antioxidant/ anti-inflammatory	Antioxidant: DPPH, ABTS, NO, FRAP, CCA, and ICA assays. Anti-inflammatory: against COX-2	[59]
	*Halorubrum* sp.	Bacterioruberin ^1^	Antioxidant	DPPH and ABTS assays	[52]
	*Haloarcula* sp. and *Halorubrum tebenquichense*	Bacterioruberin ^1^	Antioxidant	DPPH, ABTS, and FRAP assays	[53]
	*Haloferax mediterranei*	Bacterioruberin ^1^	Antioxidant	DPPH, ABTS, and FRAP assays	[54]
	*Arthrobacter* sp. G20	Crocin ^1^	Antioxidant	DPPH assay	[300]
	strain 04OKA-13-27	(3*R*)-saproxanthin ^2^	Antioxidant	Against free-radical-induced lipid peroxidation in a rat brain homogenate	[301]
Bacteria	strain YM6-073	(3*R*,2′*S*)-myxo, (3*R*,3′*R*)-zeaxanthin ^2^	Antioxidant	Against free-radical-induced lipid peroxidation in a rat brain homogenate	[301]
	strain 04OKA-17-12	(3*R*,3′*R*)-zeaxanthin ^2^	Antioxidant	Against free-radical-induced lipid peroxidation in a rat brain homogenate	[301]
	*Rubritalea squalenifaciens*	Diapolycopenedioic acid ^2^ xylosyl esters	Antioxidant	^1^O_2_ suppression model	[302]
	*Exiguobacterium acetylicum S01*	Diapolycopenedioic-acid-diglucosyl ester ^2^	Antioxidant/ anti-inflammatory	Antioxidant: DPPH assay. Anti-inflammatory: inhibition of NO production and TNF-α protein levels in LPS-induced oxidative stress in PBMC	[303]
	*Exiguobacterium acetylicum S01*	Keto-myxocoxanthinglucoside ester ^2^	Antioxidant/ anti-inflammatory	Antioxidant: DPPH assay. Anti-inflammatory: inhibition of NO production and TNF-α protein levels in LPS-induced oxidative stress in PBMC	[303]
	*Micrococcus yunnanensis*	Sarcinaxanthin ^2^	Antioxidant	^1^O_2_ suppression model	[304]
	*Micrococcus yunnanensis*	Sarcinaxanthin monoglucoside ^2^	Antioxidant	^1^O_2_ suppression model	[304]
	*Micrococcus yunnanensis*	Sarcinaxanthin diglucoside ^2^	Antioxidant	^1^O_2_ suppression model	[304]
	*Kocuria* sp. *RAM1*	Bisanhydrobacterioruberin derivative, trisanhydrobacterioruberin, and 3,4,3′,4′-tetrahydrospirilloxanthin ^1^	Antioxidant/ anti-inflammatory	Antioxidant: DPPH assay. Anti-inflammatory: hypotonic solution-induced hemolysis	[305]
	*Halobacillus halophilus* (mutant)	Hydroxy-3,4-dehydro-apo-8′-lycopene ^2^	Antioxidant	^1^O_2_ suppression model	[306]
	*Halobacillus halophilus* (mutant)	Methyl hydroxy-3,4-dehydro-apo-8′-lycopenoate ^2^	Antioxidant	^1^O_2_ suppression model	[306]
	*Planococcus maritimus*	Methyl glucosyl-3,4-dehydro-apo-8′-lycopenoate ^2^	Antioxidant	^1^O_2_ suppression model	[307]
	*Planococcus* sp. *ANT_H30*	Unidentified ^1^	Antioxidant	DPPH assay	[308]
	*Rhodococcus* sp. *ANT_H53B*	Dihydroxyneurosporene, hydroxyechinenone, and 4 unidentified ^1^	Antioxidant	DPPH assay	[308]
	*Planococcus* sp. *Eg-Natrun*	Astaxanthin and β-carotene ^1^	Antioxidant	Fenton reaction	[87]
	*Erythrobacter citreus* *LAMA 915*	Zeaxanthin, caloxanthin, nostoxanthin, adonixanthin, canthaxanthin, and erythroxanthin sulfate ^1^	Antioxidant	DPPH assay	[85]
Cyanobacteria	*Trichodesmiumemi* IMS101	Zeaxanthin, all-*trans*- and 9-*cis*-β-carotene ^1^	Antioxidant	FRAP method	[277]
	*Aphanothece microscopica Nageli*	13-*cis*-Antheroxanthin, 15-*cis-* and all-*trans*-lutein, all-*trans*-zeaxanthin, all-*trans*-cantaxanthin, all-*trans*-myxoxanthophyll, β-carotene-5,6-epoxide, all-*trans*-β-cryptoxanthin, all-*trans*-crocoxanthin, all-*trans*- and 9-*cis*-echineone, and all-*trans*- 9-*cis*-, and 13-*cis-*β-carotene ^1^	Antioxidant	Fluorescence decay resulting from the ROO· induced oxidation of the C_11_-BODIPY^581/591^ probe	[309]
	*Alkalinema aff. pantanalense*	Zeaxanthin, lutein derivatives, echinenone derivative, and unknown carotenoids ^1^	Antioxidant	^1^O_2_ suppression model	[310]
	*Cuspidothrix issatschenkoi*	Canthaxanthin, all-*trans-* and 13-*cis*-β-carotene, α-carotene derivative, and unknown carotenoids ^1^	Antioxidant	^1^O_2_ suppression model	[310]
	*Leptolyngbya-like sp.*	β-carotene oxygenated derivatives, lutein derivative, lutein, zeaxanthin, echinenone, all-*trans* and 13-*cis*-β-carotene, α-carotene derivative, and unknown carotenoids ^1^	Anti-inflammatory	Inhibition of NO production in macrophage cells	[310]
Macroalga (brown)	*Hijikia fusiformis*	Fucoxanthin ^1^	Antioxidant	DPPH assay	[311]
	*Myagropsis myagroide*	Fucoxanthin ^1^	Anti-inflammatory	Inhibition of NO in LPS-induced macrophage cells	[312]
	*Sargassum muticum*	-	Antioxidant	Evaluation of the total antioxidant capacity after supplementation in humans	[174]
	*Sargassum hemiphyllum*	Fucoxanthin and fucoidan (polysaccharide) ^1^	Anti-inflammatory	Evaluation of hepatic inflammation through modulation of leptin/adiponectin axis after supplementation in humans with NAFLD	[175]
Macroalga (green)	*Halimeda opuntia*	Unknown carotenoids ^1^	Antioxidant	DPPH assay	[313]
Macroalga (red)	*Eucheuma denticulatum*	Lutein and zeaxanthin ^1^	Antioxidant	ORAC assay	[314]
Microalga	*Dunaliella salina*	Lutein, zeaxanthin, α-carotene, all-*trans*- and 9-*cis*-β-carotene, and one unknown compound ^1^	Antioxidant/anti-inflammatory	Antioxidant: reducing capacity, chelating activity, DPPH and ^1^O_2_ suppression model. Anti-inflammatory: against COX-2 on human oral squamous carcinoma cells	[315]
	*Haematococcus pluvialis*	Astaxanthin ^3^	Antioxidant	Evaluation of oxidative damage in rats caused by high fructose consumption after supplementation of astaxanthin	[316]
	*Phaeodactylum tricornutum*	Fucoxanthin ^1^	Anti-inflammatory	Inhibition of NF-κB and NLRP3 inflammasome activation induced by the combination of LPS and ATP in bone marrow-derived immune cells and astrocytes	[317]
	*Haematococcus pluvialis*	3*S*,3′*S*-astaxanthin and 3*S*,3′*S*-astaxanthin esters ^1^	Antioxidant	^1^O_2_ suppression model	[209]
	Brown microalga	Fucoxanthin ^1^	Anti-inflammatory	Inhibition of COX-2 and iNOS expression in in macrophage cells incubated with LPS	[318]
Yeast	*Rhodosporidium paludigenum*	Carotenoids	Antioxidant	Evaluation of MDA levels in the muscle, and the activities of serum T-AOC, CAT, SOD and GPx, and hepatopancreases SOD and GPx after supplementation of shrimps with live yeast	[319]
	*Rhodotorula* sp.	Torularhodin	Antioxidant	Chemiluminescence and photochemiluminescence (Trolox) methods	[243] ^4^

^1^ Evaluation of an algae extract; ^2^ evaluation of the isolated carotenoid; ^3^ evaluation of the standard carotenoid. ^4^ In these works, the antioxidant activity of torularhodin extract produced from *Rhodotorula rubra*, which was not reported as aquatic yeast, was evaluated. However, torularhodin is produced by species of marine *Rhodotorula* [249]. ABTS, 2,2′-azino-bis(3-ethylbenzothiazoline-6-sulfonic acid; CAT, catalase; CCA, copper chelating assay; COX-2, cyclooxygenase-2; DPPH, 2,2-diphenyl-1-picrylhydrazyl; FRAP, ferric reducing antioxidant power; FRP, ferric reducing power; GPx, glutathione peroxidase; ICA, iron chelating assay, iNOS, inducible nitric oxide synthase; LPS, lipopolysaccharide; MDA, malondialdehyde; MTT, 3-(4,5-dimethylthiazol-2-yl)-2,5-diphenyl-2H-tetrazolium bromide; NAFLD, non-alcoholic fatty liver disease; NLRP3, nod-like receptor family pyrin domain containing 3; NO, nitric oxide; ORAC, oxygen radical absorbance capacity; PBMC, peripheral blood mononuclear cell; SOD, superoxide dismutase; T-AOC, total antioxidant competence; TNF-α, tumor necrosis factor-α; and Trolox, 6-hydroxy-2,5,7,8-tetramethylchroman-2-carboxylicacid.
